# WNK3 kinase maintains neuronal excitability by reducing inwardly rectifying K^+^ conductance in layer V pyramidal neurons of mouse medial prefrontal cortex

**DOI:** 10.3389/fnmol.2022.856262

**Published:** 2022-10-13

**Authors:** Adya Saran Sinha, Tianying Wang, Miho Watanabe, Yasushi Hosoi, Eisei Sohara, Tenpei Akita, Shinichi Uchida, Atsuo Fukuda

**Affiliations:** ^1^Department of Neurophysiology, Hamamatsu University School of Medicine, Hamamatsu, Japan; ^2^Department of Nephrology, Graduate School of Medical and Dental Sciences, Tokyo Medical and Dental University, Tokyo, Japan

**Keywords:** WNK3 kinase, brain development, chloride homeostasis, neuronal excitability, inwardly rectifying K^+^ channel, KCC2

## Abstract

The with-no-lysine (WNK) family of serine-threonine kinases and its downstream kinases of STE20/SPS1-related proline/alanine-rich kinase (SPAK) and oxidative stress-responsive kinase-1 (OSR1) may regulate intracellular Cl^−^ homeostasis through phosphorylation of cation-Cl^−^ co-transporters. WNK3 is expressed in fetal and postnatal brains, and its expression level increases during development. Its roles in neurons, however, remain uncertain. Using WNK3 knockout (KO) mice, we investigated the role of WNK3 in the regulation of the intracellular Cl^−^ concentration ([Cl^−^]_i_) and the excitability of layer V pyramidal neurons in the medial prefrontal cortex (mPFC). Gramicidin-perforated patch-clamp recordings in neurons from acute slice preparation at the postnatal day 21 indicated a significantly depolarized reversal potential for GABA_A_ receptor-mediated currents by 6 mV, corresponding to the higher [Cl^−^]_i_ level by ~4 mM in KO mice than in wild-type littermates. However, phosphorylation levels of SPAK and OSR1 and those of neuronal Na^+^-K^+^-2Cl^−^ co-transporter NKCC1 and K^+^-Cl^−^ co-transporter KCC2 did not significantly differ between KO and wild-type mice. Meanwhile, the resting membrane potential of neurons was more hyperpolarized by 7 mV, and the minimum stimulus current necessary for firing induction was increased in KO mice. These were due to an increased inwardly rectifying K^+^ (IRK) conductance, mediated by classical inwardly rectifying (Kir) channels, in KO neurons. The introduction of an active form of WNK3 into the recording neurons reversed these changes. The potential role of KCC2 function in the observed changes of KO neurons was investigated by applying a selective KCC2 activator, CLP290. This reversed the enhanced IRK conductance in KO neurons, indicating that both WNK3 and KCC2 are intimately linked in the regulation of resting K^+^ conductance. Evaluation of synaptic properties revealed that the frequency of miniature excitatory postsynaptic currents (mEPSCs) was reduced, whereas that of inhibitory currents (mIPSCs) was slightly increased in KO neurons. Together, the impact of these developmental changes on the membrane and synaptic properties was manifested as behavioral deficits in pre-pulse inhibition, a measure of sensorimotor gating involving multiple brain regions including the mPFC, in KO mice. Thus, the basal function of WNK3 would be the maintenance and/or development of both intrinsic and synaptic excitabilities.

## Introduction

The with-no-lysine (WNK) kinase subfamily of serine/threonine kinases is characterized by the absence of the critical ATP-binding lysine residue in the subdomain II of these kinases. Instead, the kinase function is mediated by a lysine residue in subdomain I (Xu et al., 2000). In mammals, the WNK family of kinases comprises four gene products (WNK1-WNK4) with defined spatiotemporal expression patterns (reviewed in McCormick and Ellison, 2011). Though pleiotropic in their functions, the primary physiological role ascribed to the WNK family is its phosphorylation of downstream signaling cascades integral to the maintenance of Cl^−^ homeostasis (Alessi et al., 2014). In neurons, intracellular Cl^−^ concentrations ([Cl^−^]_i_) are critical for neuronal excitability (Blaesse et al., 2009; Raimondo et al., 2017) and cell volume regulation (Akita and Okada, 2014; Huang et al., 2019). It was first reported that WNK1 phosphorylates downstream SPS1- related proline/alanine-rich kinase (SPAK) and its homolog oxidative stress-responsive kinase (OSR1) (Moriguchi et al., 2005). These kinases catalyze post-translational phosphorylation of cation-Cl^−^ co-transporters. The phosphorylation of residues Thr^212^ and Thr^217^ of human Na^+^-K^+^-2Cl^−^ co-transporter (NKCC1) increases its activation leading to intracellular Cl^−^ accumulation (Flemmer et al., 2002; Kahle et al., 2005). In striking contrast, activation of the WNK- SPAK/OSR1 cascade inhibits neuron-specific K^+^-Cl^−^ co-transporter (KCC2) by phosphorylation of residues Thr^906^ and Thr^1007^ in immature neurons (Inoue et al., 2012). Perinatally, the WNK/SPAK-OSR1 cascade exerts tight control on the relative functionalities of NKCC1 and KCC2 to set the level of Cl^−^ (Kahle et al., 2013). The Cl^−^ reported around this period is ~30 mM (Achilles et al., 2007). The significance of Cl^−^ values is that it determines the equilibrium potential (E_Cl_) of Cl^−^. Therefore, E_Cl_ values positive to resting membrane potential (RMP) cause depolarizing action of the neurotransmitter γ -aminobutyric acid (GABA) on binding to ligand-gated Cl^−^ channels (GABA_A_ receptors) in immature neurons (Ben-Ari, 2002). However, at later time points when E_Cl_ values are more negative to RMP, the classical hyperpolarizing action of GABA is observed (Yamada et al., 2004). This dependence on E_Cl_ also underlies the initial excitatory action mediated through glycine receptors (GlyR) during cortical development (Flint et al., 1998) and subsequent inhibitory neurotransmission after maturation. Thus, the regulatory changes affecting E_Cl_ prompting the remarkable switch from excitation to inhibition mediated by both GABA_A_Rs and GlyRs, represents a key event in the developmental trajectory of the brain. Accordingly, this posits the role of the WNK-SPAK/OSR1 cascade to be of utmost importance during brain development (Watanabe and Fukuda, 2015).

More recent investigations on the role of WNK kinases in neurons have confirmed WNK1 kinase functioning as a Cl^−^ sensor (Piala et al., 2014). The binding of Cl^−^ inactivates WNK1 by inhibiting its autophosphorylation. Recent experiments have revealed that WNK1 forms a physical complex with KCC2 (Friedel et al., 2015) and confirmed inhibitory phosphorylation of C-terminal threonine residues (Thr^906/1007^) restricting KCC2 activity. Furthermore, constitutive phosphorylation of these threonine residues (KCC2^T906E/T1007E^) in a mice model caused early postnatal death due to respiratory arrest (Watanabe et al., 2019). These accumulating evidences underline the importance of WNK1 in normal brain development. Surprisingly, given its importance, the only pathology of nervous tissue associated with WNK1 are rare forms of hereditary sensory and autonomic neuropathy (Lafreniere et al., 2004).

On the contrary, mounting evidence from clinics indicates another member of the WNK family, WNK3, to be associated with diverse pathologies of the central nervous system. In brief, the expression of WNK3 transcripts was found to be significantly higher in the prefrontal cortex (PFC) area of subjects with schizophrenia (Arion and Lewis, 2011). Furthermore, an increase in WNK3 was reported from dispersed granule cells of subjects presenting a pathology of hippocampal sclerosis associated with mesial temporal lobe epilepsy (Jeong et al., 2018). Incidentally, WNK3 loci are on the X-chromosome (Holden et al., 2004) and within the critical linkage zone associated with several monogenic intellectual disability disorders. In fact, one report (Qiao et al., 2008) correlates microdeletion of this region Xp11.22 with autistic behavioral phenotype. More recently, using exome sequencing analysis, multiple pathogenic missense variants for WNK3 were identified (Küry et al., 2022). All the individuals were affected with developmental delay and showed intellectual disability with 38% of them exhibiting epilepsy. Surprisingly, all these rare variants are predicted to cause loss of function of WNK3. In the fetal brain, strong expression of WNK3 mRNA transcripts was observed. Subsequently, expression was observed to be weak around postnatal day 7 with transcripts almost undetectable by postnatal day 10. Following this period, the WNK3 expression increased again plateauing around postnatal day 21 (Kahle et al., 2005). This increase of WNK3 transcripts from postnatal day 10 to 21 leads to earlier reports drawing parallels with the KCC2 expression pattern (Lu et al., 1999; Kahle et al., 2005, 2013). However, a more recent report (Küry et al., 2022) indicates that postnatal period WNK3 levels are significantly lower in comparison to their fetal expression levels. In addition, it has also been observed that the expression level of WNK1 is 10-fold higher than that observed in WNK3 postnatally (Heubl et al., 2017). Therefore, comparisons of expression patterns of WNK3 and KCC2 commencing from early development to the postnatal period actually are divergent (Küry et al., 2022). The earliest investigation (Kahle et al., 2005) probing the physiological role of WNK3 earmarks it as a key effector of Cl^−^-dependent volume regulation. This result was supported by recent experimental findings using stroke models as below. While activated WNK3 function exacerbates brain injury after cerebral hemorrhage (Wu et al., 2020), either inhibition of WNK3 function (Begum et al., 2015) or deletion of WNK3-SPAK complex reduced brain damage and accelerated neurological recovery (Zhao et al., 2017). Furthermore, its pleiotropic nature was revealed by biochemical investigations reporting WNK3-dependent suppression of neuronal splicing factor Rbfox1 (Lee et al., 2012). Interestingly, Rbfox1 is a nodal point in the signaling pathways altered in autistic brains (Voineagu et al., 2011) and implicated in synaptic dysfunctions (Gehman et al., 2011; Lee et al., 2016).

Based on these findings, it is probable that WNK3 plays an important role during fetal development and postnatally affects neuronal excitability during brain development. We therefore used a constitutive WNK3 knockout (KO) mouse (Oi et al., 2012) and investigated this possibility. Our results suggest that WNK3 in pyramidal neurons plays a more critical role in the maintenance of neuronal excitability by reducing resting membrane K^+^ conductance and increasing the number of excitatory synaptic inputs. It also impacts ionic plasticity albeit weakly by regulating intracellular Cl^−^ homeostasis. The impact of these developmental changes in membrane and synaptic properties of pyramidal neurons in WNK3 KO mice together with yet uninvestigated changes in other neuronal subtypes and cells alters information processing in the mPFC. Together, these changes manifested as behavioral deficits in pre-pulse inhibition, a measure of sensorimotor gating involving multiple brain regions including the mPFC. Thus, the basal function of WNK3 would be the maintenance and/or development of both intrinsic and synaptic excitabilities.

## Materials and methods

### WNK3 knockout mice

The WNK3 knockout mice were generated as explained earlier elsewhere (Oi et al., 2012). In brief, the Cre-Lox recombination system was used to target the WNK3 gene for deletion. Experimental mice were generated by mating WNK3^+/−^ heterozygous female mice with male (>10 weeks) WNK3^−/−^ mice to obtain both constitutive knockout (KO) and wild-type (WT) littermates. All experiments were planned to minimize the number of animals used. The procedures used were in accordance with the guidelines issued by the Hamamatsu University School of Medicine and were approved by the Committee for Animal Care and Use (No. 2018048).

### Slice preparation

Postnatal day (P) 21–27 old male mice comprising WNK3 KO and WT littermate were anesthetized with 50–90 mg/Kg intraperitoneal injection (i. p.) of pentobarbital and cardially perfused with ice-cold oxygenated, modified artificial cerebrospinal fluid (ACSF; in mM, 220 sucrose, 2.5 KCl, 1.25 NaH_2_PO_4_, 2.0 MgSO_4_, 0.5 CaCl_2_, 26.0 NaHCO_3_, and 30.0 glucose at pH 7.4). After perfusion, the mice were decapitated, and the brains were quickly dissected out. Coronal slices containing the prefrontal cortex with a thickness of 350 μm were prepared in modified ACSF using a vibratome (Campden Instruments, Loughborough, Leicestershire, UK). Slices were allowed to recover for 60 min on nylon meshes (with 1 mm pores) placed on dishes and submerged in standard ACSF consisting of (in mM) 126 NaCl, 2.5 KCl, 1.25 NaH_**2**_PO_4_, 2.0 MgSO_4_, 2.0 CaCl_2_, 26.0 NaHCO_3_, and 20.0 glucose, with osmolarity value of 310-312 mOsm and saturated with 95% O_2_ and 5% CO_2_ at room temperature (RT).

### Solutions for recordings

All recordings were performed in standard ACSF as bath solution at RT. The electrode was filled with solutions of different compositions depending on the experiments performed. Current-clamp recordings for evaluation of passive and active properties were performed using an internal solution containing (in mM) 140.0 potassium methane sulfonate (CH_3_SO_3_K), 10.0 KCl, 2.0 MgCl_2_, 10.0 HEPES, 3.0 Na_2_-ATP, 0.2 Na-GTP, and 1.0 EGTA with pH adjusted to 7.3 with KOH and osmolarity value of 310 mOsm. The liquid junction potential (LJP) was calculated to be 4.8 mV and was corrected during recordings. Recordings were performed in the presence of bath-applied ionotropic glutamate receptor blockers, 10 μM 6-cyano-7-nitroquinoxaline-2,3-dione (CNQX), 50 μM 2-amino-5-phosphonopentanoic acid (D-AP5), and 50 μM picrotoxin (PTX, Tocris), a GABA_A_ receptor antagonist. Miniature excitatory post-synaptic currents (mEPSCs) were recorded using the same internal solution with bath-applied 50 μM PTX and 0.5 μM TTX (Tetrodotoxin, Wako). The Ba^2+^-sensitive inward rectifier currents were isolated in the presence of 200 μM BaCl_2_-2H_2_O and 1 μM TTX. In a subset of experiments for recording inward rectifier potassium conductance (IRK), the active form of WNK3 kinase (Eurofins) was dissolved in the CH_3_SO_3_K-based normal Cl^−^ internal solution. The final concentration of the WNK3 active peptide fragment in the internal solution was 3.4 ng/μl. In addition, to investigate the role of KCC2 function on IRK conductance, we used KCC2 antagonist [(dihydroindenyl)oxy] acetic acid (DIOA, Sigma) dissolved in DMSO. A final concentration of 30 μM in the internal solution was used. This strategy was utilized to reduce the off-target effects of DIOA, as a recent report (Chi et al., 2021) suggests the binding of DIOA closer to the CTD region of KCC2 transmembrane assembly. After whole-cell patch-clamp recordings, we allowed a period of 20 min for DIOA action all the while monitoring for stable recording. The effect of antagonizing KCC2 function on WT neurons was evaluated by a subsequent application of Ba^2+^ block and IRK current recordings. To investigate if KCC2 membrane expression and stability exerted an effect on IRK currents as reported earlier (Goutierre et al., 2019), we incubated acute mPFC slices from both WT and KO mice with selective KCC2 activator drug CLP 290 (30 μM) for 1 h at 30–32°C. This was followed by recording Ba^2+^-sensitive IRK currents. For recording miniature inhibitory post-synaptic currents (mIPSCs), a high [Cl^**−**^] internal solution of the following composition (in mM) was used: 150.0 CsCl, 2.0 MgCl_2_, 10.0 HEPES, 3.0 Na_2_-ATP, 0.2 Na-GTP, and 1.0 EGTA with pH adjusted to 7.3 with CsOH and osmolarity of 305 mOsm. The E_**Cl**_ was calculated to be −3.9 mV. The corresponding calculated LJP value of 4.6 mV was corrected during recordings. The bath solution contained 10 μM CNQX, 50 μM D-AP5, and 0.5 μM TTX. For estimation of [Cl^**−**^]_i_, gramicidin-perforated patch-clamp recordings were performed. The electrode solution comprised 150.0 mM KCl and 10.0 mM HEPES with pH adjusted to 7.3 using KOH. On the day of the experiment, gramicidin (Sigma) was dissolved in solvent methanol to obtain a stock. Subsequently, aliquots of this stock were used every 2 h and added to a fresh aliquot of KCl-based internal solution with a final concentration of 50 μg/ml gramicidin in the electrode solution. To the bath solution, excitatory synaptic blockers CNQX (10 μM) and D-AP5 (50 μM) were added. In addition, TTX (1 μM) and CGP55845 (3 μM) were applied to block Na^+^-dependent action potentials and GABA_B_ receptors, respectively. The calculated LJP of −3.6 mV was not corrected, as it was negated by the E_K_ of approximately +4 mV owing to the higher K^+^ ion concentration in the electrode solution in comparison to that of the cytosol (Kim and Trussell, 2007).

### Whole-cell patch-clamp recordings

Slices thus obtained were then transferred to an imaging chamber on the stage of an upright microscope (BX51WI; Olympus Tokyo, Japan) and continuously perfused with oxygenated standard ACSF at a flow rate of 2 mL/min at RT. The electrode resistance ranged from 3 to 5 MΩ. Whole-cell patch-clamp recordings were made from layer V pyramidal neurons of the medial prefrontal cortex (mPFC). The neurons were identified based on their pyramidal shape and long apical dendrites. The soma was located at a distance of >500 μm from the medial midline. Voltage changes were recorded by a multiclamp 700B amplifier (Axon Instruments, Sunnyvale, CA, USA) with a Bessel pre-filter at 5 kHz and digitized at 25 kHz using a Digidata 1440A data-acquisition system (Axon instruments, Sunnyvale, CA, USA).

For current-clamp recordings to study passive and active properties, step current injections of Δ 20 pA starting from −60 pA to +540 pA with a duration of 1,000 ms were made. A short pulse (2 ms) protocol for eliciting single action potential (AP) was used for the evaluation of the AP waveform. The bridge balance circuit was applied while recording the voltage changes. Voltage clamp recordings were performed for evaluating synaptic physiology. In brief, both mIPSCs and mEPSCs were recorded at a holding voltage (**V**_**H**_) of −70 mV using the aforementioned internal solutions. Voltage steps for isolating inward rectifying K^+^ (IRK) currents were of 500 ms duration starting from **V**_**H**_ of −50 mV to −120 mV with a step size of Δ10 mV. The series resistance (**R**_**s**_) compensation circuit was applied at 70% during recordings. Recordings from neurons with R_s_ exceeding 25 MΩ were not used. The GABA reversal potential (E_GABA_) measurements were performed from layer V pyramidal neurons at P 21 by using the gramicidin-perforated patch-clamp technique. In brief, the tip of the patch electrodes was filled by capillary action with a gramicidin-free KCl-based solution as described above. The gramicidin-containing electrode solution was back-filled. This allowed the formation of tight gigaseal >8 GΩ. In 1–1.15 h, the gramicidin diffused forming perforations on the neuronal membrane such that Rs changed to <100 MΩ. An additional 10 min were allowed to confirm a stable recording condition. Starting from a set voltage of −60 mV (**V**_**H**_), a voltage ramp of 300 ms long from −90 mV to −30 mV was applied. Matched with the ramp protocol, a 300 μM GABA puff was applied for a duration of 1 s. The holding current levels immediately before and after the voltage ramp were unchanged. The R_s_ compensation at 70% was applied. Each measurement was repeated two times following an interval of 5 min, and if the differences in the obtained E_GABA_ values were > ±1 mV, the data were discarded.

### Electrophysiological analysis

The data were acquired using a pCLAMP suite comprising Clampex 10.7 for acquisition, and analysis was performed using Clampfit 10.4 software. In brief, to evaluate the input resistance (**R**_**In**_) of neurons recorded, small hyperpolarizing current injections of 1,000 ms duration were applied starting from −60 pA. The voltage responses obtained were plotted to their corresponding current injection amplitudes to generate current-voltage relationships. The data points were subsequently fitted by linear regression analysis. The calculated slope value indicated the **R**_**In**_ of the recorded neuron. The membrane time constant (**τ**_m_) was estimated by the single exponential fitting of the membrane charge phase in response to the −40 pA current injection. The cell capacitance (C_m_) value was thereafter calculated from the relationship (**τ**_m_ = C_m_R_m_). The rheobase current, i.e., the current injection at which the neuron fired a single AP was estimated by applying depolarizing current steps 1,000 ms long with step increments of 20 pA. Single APs were elicited by the application of a short (2 ms) depolarizing pulse protocol, and its waveform parameters like AP amplitude, half-width, maximum rise, and decay slope were analyzed. Resting membrane potential (RMP) was calculated from traces at zero current injection (I = 0) levels preceding the application of the short pulse protocol. AP amplitude was calculated from RMP to AP peak voltage. AP threshold voltages were determined by phase plane plot analysis. Furthermore, repetitive AP firing elicited by depolarizing current steps was used to investigate the spike number output to current injection relationship (I-O curves) of recorded neurons. In addition, a comparison of frequency-current injection (F/I) plots (number of spikes at 1X, 2X, and 3X rheobase currents) was used to evaluate gain. Adaption parameters of repetitive firing were compared between groups at 3X rheobase currents. In brief, all parameters were compared by normalizing to the first AP evoked during a spike train. AP amplitude values were calculated from threshold voltage to peak amplitudes for adaptation parameters. The E_GABA_ values were calculated as voltage values at which the GABA_A_ receptor-mediated current reverses direction and ascertained the intersection of current responses' before and during 300 μM GABA puff application. The voltage shifts due to 30% of uncompensated R_s_ were corrected to calculate the exact values. For each cell, the E_GABA_ values were calculated from an average of two repeated measurements. In addition, approximate internal chloride concentrations were back-calculated using the Nernst equation.

Analysis of mIPSC events was performed using the threshold-based event detection suite of clampfit. A single epoch of 60 s was selected for analysis. The threshold was set at three times the standard deviation (3 S.D.) of the baseline noise. Likewise, an analysis of mEPSCs was performed. Analysis of IRK currents was performed by subtracting current responses in the presence of Ba^2+^ block from control responses to voltage commands. I-V plots were constructed for different treatments and compared.

### Immunoblotting

Under deep anesthesia, mPFC was removed, homogenized, and lysed in a buffer containing 50 mM Tris-HCl (pH 7.5), 150 mM NaCl, 1% (v/v), Triton X-100, 5 mM EDTA, protease inhibitors (Roche complete protease inhibitor cocktail tablets, 1 tablet per 50 ml), and phosphatase inhibitor cocktail 3 (P0044, Sigma). Tween–tris-buffered saline (TTBS) contained 50 mM Tris-HCl (pH 7.5), 0.15 M NaCl, and 0.1% (v/v) Tween 20. A 2x SDS sample buffer consisted of 0.1 M Tris-HCl, pH 6.8, 4% SDS, 20% glycerol, 1% (v/v) 2-mercaptoethanol, and 0.01% bromphenol blue. After centrifugation at 12,000 g for 10 min at 4 °C, supernatants were collected, and protein concentration was determined using the Bradford method with bovine serum albumin (BSA) as the standard. Aliquots of 30 μg of protein were mixed with sample buffer and boiled at 95 °C for 10 min. Samples were resolved by SDS-polyacrylamide gel electrophoresis and transferred to polyvinylidene difluoride membranes. The membranes were incubated for 1 h with blocking buffer containing 3% (w/v) BSA in TTBS and then immunoblotted in blocking buffer with indicated primary antibodies overnight at 4°C. Blots were probed with antibodies to WNK1 phospho-Ser-382, total WNK1, SPS1-related proline/alanine-rich kinase (SPAK) phospho-Ser-373, total SPAK, total oxidative stress response 1 (OSR1), NKCC1, NKCC1 phospho-Thr-206, KCC2, and actin, and then detected using HRP-conjugated secondary antibodies and an ECL kit (Amersham Biosciences). The amount of protein loaded was monitored by immunoblotting for actin. The KCC2 antibody (1:1,000) was purchased from Millipore and the actin antibody (1:10,000) from Sigma. NKCC1 phospho-Thr-206 antibody (1:400) was previously reported (Yang et al., 2010). The remaining antibodies used for Western blots were raised in sheep and affinity-purified on the appropriate antigen and were kindly provided by Dr. Alessi, University of Dundee. Antibodies prepared in sheep were used at a concentration of 1 μg/ml. The incubation with phosphorylation site-specific sheep antibodies was performed with the addition of 10 μg/ml of the non-phosphorylated peptide antigen used to raise the antibody. In order to evaluate the phosphorylation levels of KCC2 at the residue Thr1007, cortical tissue was homogenized in ice-cold lysis buffer containing 50 mM Tris-HCl, pH 7.5, 150 mM NaCl, 1% Nonidet P40, and 0.5% sodium deoxycholate, supplemented with protease inhibitor mixture (Roche) and phosphatase inhibitor cocktail 3 (Sigma-Aldrich). The lysates were centrifuged at 12,000 g for 10 min at 4°C. Immunoprecipitation was performed using the immunoprecipitation kit (Roche) according to the manufacturer's protocol. Briefly, 3.5 mg of lysate was precipitated with 5 μg of phospho-specific KCC2 antibody (Dundee) in the presence of 10 μg of the dephosphorylated form of the phosphopeptide antigen for 1 h at 4°C followed by incubation with protein G-Sepharose overnight at 4°C. The immunoprecipitates were washed in triplicate with the above buffer solution before being diluted in the SDS sample buffer and analyzed by immunoblotting using the KCC2 antibody. Relative intensities of immunoblot bands were determined using densitometry analysis with ImageJ software, and expression levels were compared between the groups as fold changes against actin. The phosphorylated KCC2 band intensity was normalized to the KCC2 band intensity.

### Behavioral test

A series of behavioral assays were conducted with male mice aged 8–10 weeks. The mice weighed 20 ± 2 g [*n* = 8(WT) and 4(WNK3KO)] on average at the beginning of the tests.

#### Open field test

The apparatus used consisted of a square base (42 × 40 cm) surrounded by a 22 cm wall. The testing arena was divided into 16 squares. The “border” is defined as the 12 outer periphery squares and the “center” as the 4 central squares. Each mouse was placed individually in the center of the open-field apparatus. Testing was conducted over 5 min and recorded with the detection of multiple body points (nose, middle, and tail) of the mice using a video tracking system (SMART v3.0; Panlab/Harvard Apparatus, Barcelona, Spain). The walls and floors of the apparatus were cleaned thoroughly with 10% ethanol between tests. The total distance and the time spent in the center were calculated.

#### Social interaction and social recognition memory test

The three-chamber test of social interaction and social novelty recognition was performed as described previously (Silverman et al., 2010) with a slight modification. The apparatus consisted of three compartments (40 × 20 × 22 cm) made of clear plexiglass, each separated by side doors. The social communication activities were also measured by using the SMART video tracking system. The task included four sessions. In the first session, a test mouse was habituated to the center chamber for 5 min. In the second session, a test mouse was allowed to explore all three chambers for 10 min. Before the third session, an unfamiliar mouse (Stranger 1, S1) was placed in a steel cage (11 cm D × 25 cm H) enclosure in the left or right chamber, chosen randomly to avoid side preference. In the third session, the subject mouse was allowed to explore all three chambers and cages. Social interaction was determined by measuring the length of time spent by the test mouse exploring the chamber holding the unfamiliar Stranger 1 vs. the empty chamber (E). To measure the social novelty, a new unfamiliar mouse (Stranger 2, S2) was placed in the steel enclosure in the previously empty chamber, and the existing unfamiliar Stranger 1 was retained in the same chamber. The experimental mouse was left to freely explore the chambers for 10 min. The amount of time spent by the test mouse for exploring the chamber containing the familiar Stranger 1 and the novel unfamiliar Stranger 2 mouse was measured to determine the social memory or social novelty. The preference index (%) was calculated from the time spent as (S1 – E)/(S1 + E) × 100 for social interaction, where E denotes empty cage, and (S2 – S1)/(S2 + S1) × 100 for social novelty recognition. The apparatus was cleaned with 70% ethanol between each trial.

#### Pre-pulse inhibition test

Pre-pulse inhibition (PPI) experiments were conducted with a Panlab System (San Diego Instruments, Inc., San Diego, CA). Each PPI session began with a 5 min habituation period (no stimuli). The test sessions consisted of a series of 5 pulse-only trials (120 dB) for habituation purposes followed by six repetitions of a 9 trial block for a total of 59 trials. The nine trial types included one blank trial (no stimuli), two startle pulse (110 and 120 dB; 40 ms) trials, two pre-pulse (74 and 78 dB; 40 ms) trials, and four pre-pulse + pulse trials [74/78 dB (40 ms) + 110/120 dB (40ms)] and were pseudo-randomized with a 10–20s inter-trial variable interval. All tests were fully computerized by the PACKWIN v2.0 software package (Panlab/Harvard Apparatus, Barcelona Spain). PPI was calculated as a percent score (% PPI) using the following formula: 1–(average startle response on prepulse + pulse trials/average startle response on pulse-alone trials) × 100.

### Statistical analysis

For analysis of data obtained by patch-clamp experiments, statistical analysis was performed using IBM SPSS Ver.23 software. In brief, data were tested for normality using the *Kolmogorov–Smirnov* (K-S) test, upon both non-significant K-S statistic and *Levene's statistic* confirming normal distribution and equality of variance, respectively. Independent samples *t-test* was used for comparison of the two groups. However, if the K-S statistic confirmed non-normal distribution, then for two groups *Mann–Whitney U test* and for multiple groups *Kruskal–Wallis test* followed by *post-hoc* stepwise stepdown test was employed. Distributions of miniature events from WT and WNK3 KO were compared by the K-S test. Data are presented as the mean ± standard error of the mean (SEM). Statistical significance was presented using the following rules: ^*^P < 0.05, ^**^P < 0.01, ^***^P < 0.001, and ns represents not significant.

## Results

### WNK3 loss depolarizes GABA reversal potential in layer V pyramidal neurons

**Figure 1:**
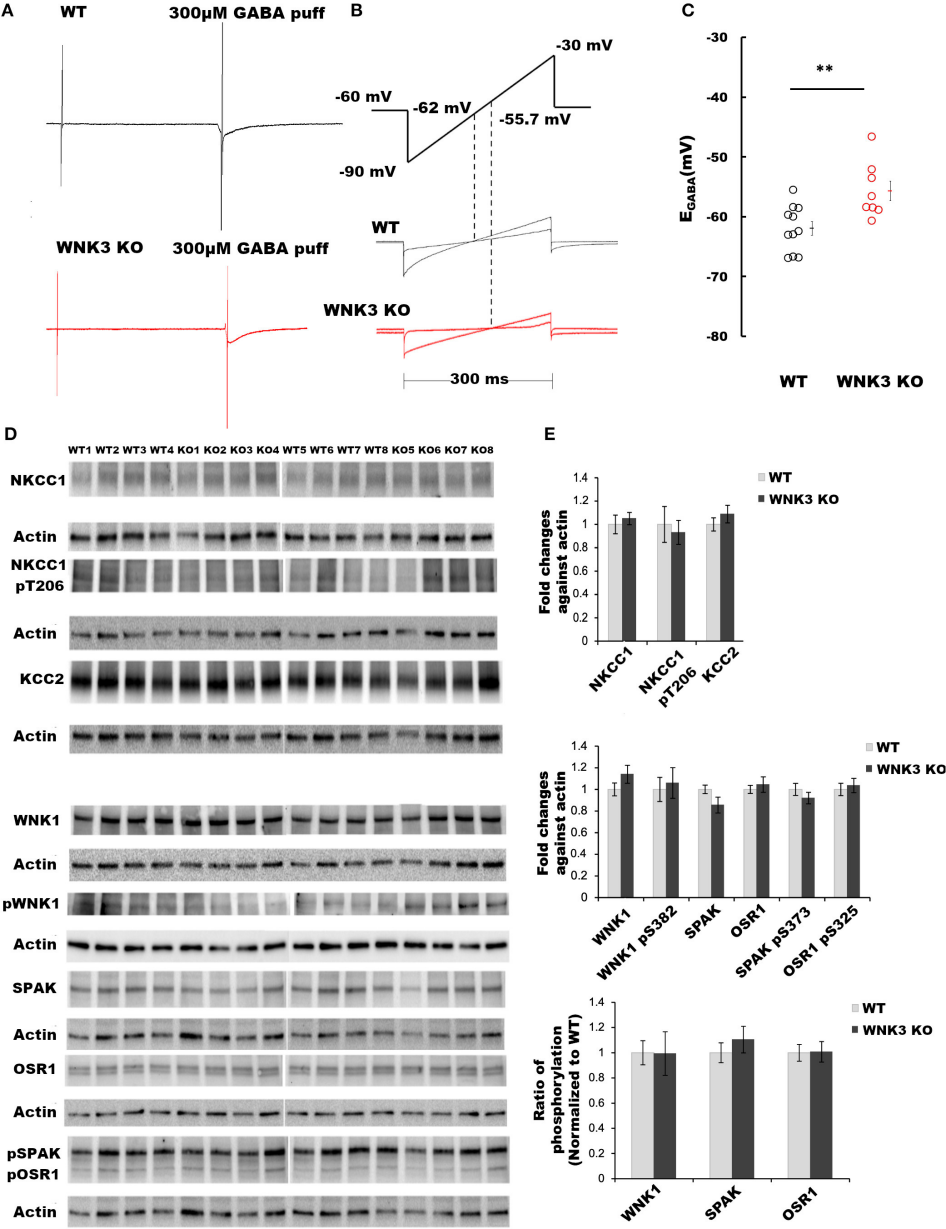
WNK 3 loss depolarizes GABA reversal potential in layer V pyramidal neurons at postnatal day 21. **(A)** Gramicidin-perforated patch recordings showing current traces in WT (black) and WNK3 KO neurons (red). The voltage ramps were applied before and during 300 μM GABA application to determine the GABA reversal potential (E_GABA_). The rise and decay phase of the current response to GABA puff application were unchanged by the ramp protocol **(B)**. Voltage ramp protocol was performed to determine E_GABA_ (upper panel). The holding voltage was clamped at −60 mV. Two voltage ramps starting from −90 mV to −30 mV of 300 ms duration in the absence and presence of GABA (1s) were applied. The middle and lower panels show representative current responses to these two ramp protocols in WT (black) and WNK3 KO neurons (red), respectively. Dashed lines indicate the corresponding voltage level at which the two responses intersect indicating the E_GABA_ values. **(C)** Quantitative analysis of E_GABA_ of layer V pyramidal neurons from the mPFC at P 21. WT (black open circles) and WNK3 KO (red open circles) indicate individual data points. The mean values are indicated with solid bars next to the data points (WT = −61.95 ± 1.16 mV, *n* = 11 cells, 6 mice; WNK3 KO = −55.70 ± 1.64 mV, *n* = 8 cells, 6 mice; *t-*test ** *P* < 0.01). **(D)** Immunoblots for evaluating changes in WNK1-SPAK/OSR1 cascade and cation- Cl^**−**^ cotransporters. Protein blots for total NKCC1, pNKCC1, and total KCC2 protein levels (upper panel) followed by protein blots of WNK1, pWNK1, total SPAK, total OSR1, and pSPAK1/pOSR1 (lower panel) are shown. Actin was used as a loading control. **(E)** Densitometric analysis for NKCC1, pNKCC1, and KCC2 (upper panel) and WNK1, pWNK1, total SPAK/OSR1, and pSPAK/pOSR1 (lower panel) showed no significant differences between WT (Gray bars) and WNK3 KO (Black bars) at P 21. Intensities were normalized to WT levels. *N* = 8 mice in each group. Error bars represent SEM.

The primary function of the WNK subfamily of kinases is the regulation of Cl^**−**^ homeostasis. The expression levels of WNK3 kinase peak at P21 (Kahle et al., 2005). Therefore, using acute slice preparation at P21, we performed gramicidin-perforated patch-clamp recordings from layer V pyramidal neurons to understand the effect of WNK3 knockout on E_GABA_ values. We used a ramp protocol to determine the E_GABA_ values. Examples of current recordings from WT (black) and WNK3 KO (red) neurons to voltage ramps (−90 mv to −30 mV, 300 ms) in the absence and the presence of 300 μM GABA are shown in Figure 1A. The representative ramp responses from WT and WNK3 neurons, with corresponding intersection voltage values marked on the ramp protocol, are illustrated in Figure 1B. Statistical comparisons of recorded E_GABA_ values indicate a significant depolarization shift of reversal potentials by 6 mV in WNK3 KO neurons (WT = −61.95 ± 1.16 mV, *n* = 11 cells, 6 mice; WNK3 KO = −55.70 ± 1.64 mV, *n* = 8 cells, 6 mice; *t-test, P* =*P* = 0.0050) as shown in Figure 1C. This depolarization corresponds to a 4 mM increase in resting [Cl^**−**^]_i_ levels in WNK3 KO pyramidal neurons when compared to WT neurons. The observed increase in [Cl^**−**^]_i_ levels following the loss of WNK3 may correspond to a compensatory increase in the activity of WNK1 as observed in the mouse kidney (Mederle et al., 2013). The possibility of such occurrences has also been proposed in mature neurons (Heubl et al., 2017). Incidentally, in mature neurons, the mRNA transcripts of WNK1 and WNK3 are more abundant than other isoforms (Heubl et al., 2017). Therefore, to confirm this possibility, immunoblotting experiments evaluating compensatory activation of WNK1-SPAK/OSR1 cascade onto NKCC1 and KCC2 were performed. However, the phosphorylation levels of residue Thr-206 (pNKCC1), a marker for increased NKCC1 function (Rinehart et al., 2009; Yang et al., 2010), were similar between groups. Total KCC2 protein levels were also observed to be not significantly different (Figures 1D,E). We observed no significant difference in the expression levels of total WNK1 levels (Figures 1D,E) between the WT and WNK3 KO groups. The activation of WNK1 requires autophosphorylation of Ser-382 (De Los Heros et al., 2014). The phosphorylation levels of residue Ser-382 (pWNK1) between WT and WNK3 KO were observed to be similar. Another possibility would be that loss of WNK3 directly alters the phosphorylation status of downstream signaling kinases SPAK/OSR1. We therefore evaluated changes in total SPAK/OSR1 protein levels, as well as phosphorylated SPAK/OSR1 (Inoue et al., 2012). The total protein levels of SPAK/OSR1 were not significantly altered in the KO mice (Figures 1D,E). In addition, phosphoantibodies against Ser-373 (pSPAK) and Ser-325 (pOSR1) revealed no significant differences (Figures 1D,E).

**Figure 2:**
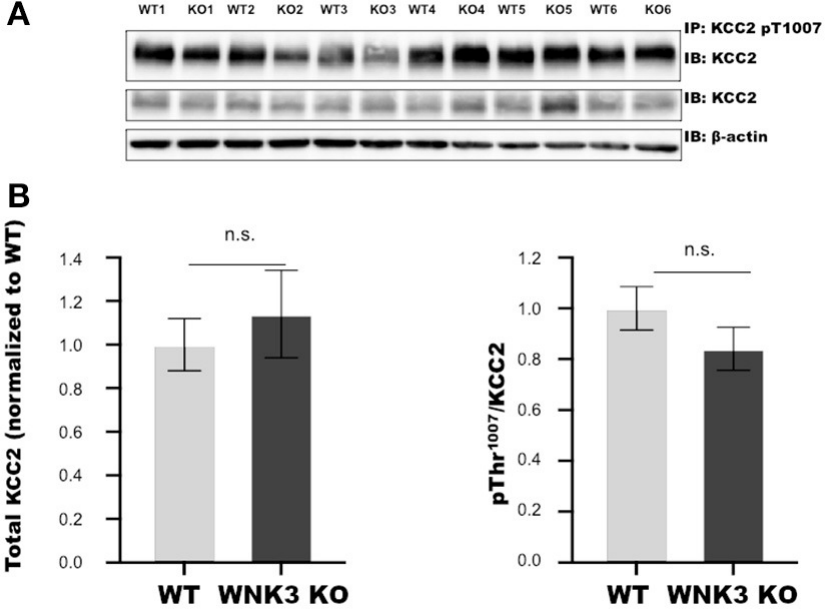
WNK 3 KO mice cortex show no change in KCC2 pT1007 levels at postnatal day (P) 21 in comparison to WT littermates. **(A)** Immunoblots for pThr1007 KCC2 and total KCC2 protein levels following immunoprecipitation from postnatal day 21 cortex. Actin was used as a loading control. **(B)** Densitometric analysis for total KCC2 (left panel) and pThr^1007^/KCC2 (right panel) showed no significant differences between WT (Gray bars) and WNK3 KO (Black bars) at P 21. Intensities were normalized to WT levels. *N* = 6 mice in each group. Error bars represent SEM.

The WNK- SPAK/OSR1 signaling cascade also exerts a regulatory role on KCC2 function by inhibitory phosphorylation of C-terminal residue Thr1007. The progressive downregulation of this posttranslational modification in the course of development is essential for mouse survival (Watanabe et al., 2019). Alterations in this programmed downregulation in pT1007 KCC2 levels may contribute to the depolarizing shift of E_GABA_ (Figures 1B,C) in WNK3 KO neurons. We therefore evaluated if the loss of WNK3 all throughout the fetal and postnatal developmental period disrupts this phenomenon. We immunoprecipitated pT1007 KCC2 using a phospho-specific antibody as reported earlier (Zhang et al., 2016) followed by immunoblotting. We observed no significant differences in the phosphorylation levels of Thr1007 residue in the C-terminal region of native KCC2 between the WT and WNK3 KO groups (Figures 2A,B). Together, these results suggest that the ~4 mM enhancement of resting [Cl^**−**^]_i_ level was not dependent on compensatory overactivation of the WNK1-SPAK/OSR1 cascade.

### Enhanced resting membrane conductance in WNK3 KO neurons alters passive membrane properties

**Figure 3:**
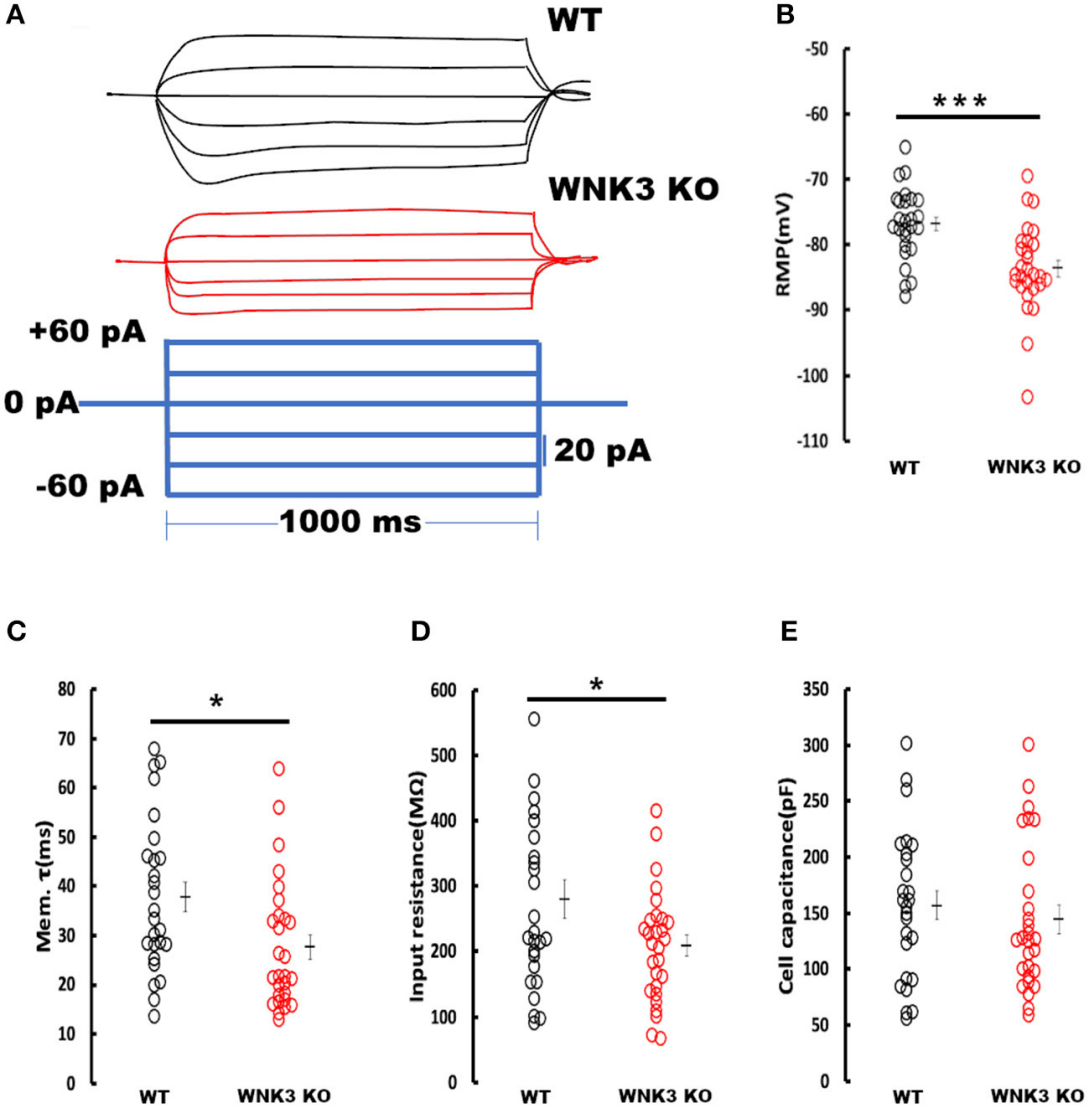
Enhanced resting membrane conductance in WNK3 KO neurons alter passive membrane properties. **(A)** Representative current-clamp recordings at small hyperpolarizing and depolarizing current steps. Step current injections of Δ 20 pA starting from −60 pA to +40 pA (lower panel, blue current step) and corresponding membrane voltage changes recorded from WT (upper panel, black traces) and WNK3 KO (middle panel, red traces) neurons are illustrated. The duration of the step pulse was 1,000 ms. **(B)** Quantitative analysis of RMP values indicate significantly hyperpolarized membrane potential in WNK3 KO neurons (WT = −76.81 ± 1.07 mV, *n* = 26 cells, 7 mice; WNK3 KO = −83.60 ± 1.26 mV, *n* = 28 cells, 7 mice; *t-*test *** *P* < 0.001). **(C)** Comparison of membrane time constant (**τ**_m_) values are significantly decreased (WT = 37.93 ± 3.05 ms; WNK3 KO = 27.68 ± 2.48 ms; *Mann–Whitney U* test, * *P* < 0.05). **(D)** Quantitative analysis of input resistance of layer V pyramidal neurons from the mPFC. The input resistance of pyramidal neurons is significantly reduced in the WNK3 KO group (WT = 280.4 ± 29.15 MΩ; WNK3 KO = 209.03 ± 16.02 MΩ; *t-*test, *P < 0.05). **(E)** Plots of calculated cell capacitance values were similar in WT and WNK3 KO neurons (WT = 156.86 ± 12.98 pF; WNK3 KO = 144.31 ± 12.39 pF; *Mann–Whitney U* test*, ns*). Individual data points are plotted as WT (black open circles) and WNK3 KO (red open circles). The mean values are indicated with solid bars next to the data points. Error bars represent SEM.

We next investigated the effect of WNK3 loss on passive membrane properties of layer V pyramidal neurons in the mPFC. Representative voltage responses from WT and WNK3 KO neurons to small hyperpolarizing and depolarizing current steps are depicted in Figure 3A. These traces indicate a notable reduction in voltage responses to the same current injection intensities in WNK3 KO neurons. A comparison of membrane potential values calculated from zero current injection (I = 0) traces exhibited a significantly hyperpolarized RMP in WNK3 KO neurons (WT = −76.81 ± 1.07 mV, *n* = 26 cells, 7 mice; WNK3 KO = −83.60 ± 1.26 mV, *n* = 28 cells, 7 mice; *P* = 0.000158) as plotted in Figure 3B. The membrane time constants estimated by the single exponential fitting of the charging phase were observed to be significantly decreased (WT = 37.93 ± 3.05 ms; WNK3 KO = 27.68 ± 2.48 ms; *P* = 0.011) as plotted in Figure 3C. In addition, estimation of the input resistance of layer V pyramidal neurons revealed a significant reduction in the WNK3 KO in comparison to WT (WT = 280.4 ± 29.15 MΩ; WNK3 KO = 209.03 ± 16.02 MΩ; *P* =0.038, Figure 3D). However, the calculated cell capacitance values calculated using the relationship (C_m_ = R_m_/**τ**_m_) showed no difference between the neurons of WT and WNK3 KO mice (WT = 156.86 ± 12.98 pF; WNK3 KO = 144.31 ± 12.39 pF; *P* =0.359, Figure 3E). These results indicate enhancement of ionic conductance at resting states, leading to hyperpolarization of RMP and significant reduction of input resistance.

### WNK3 kinase regulates classical inwardly rectifying potassium channels through kinase activity

**Figure 4:**
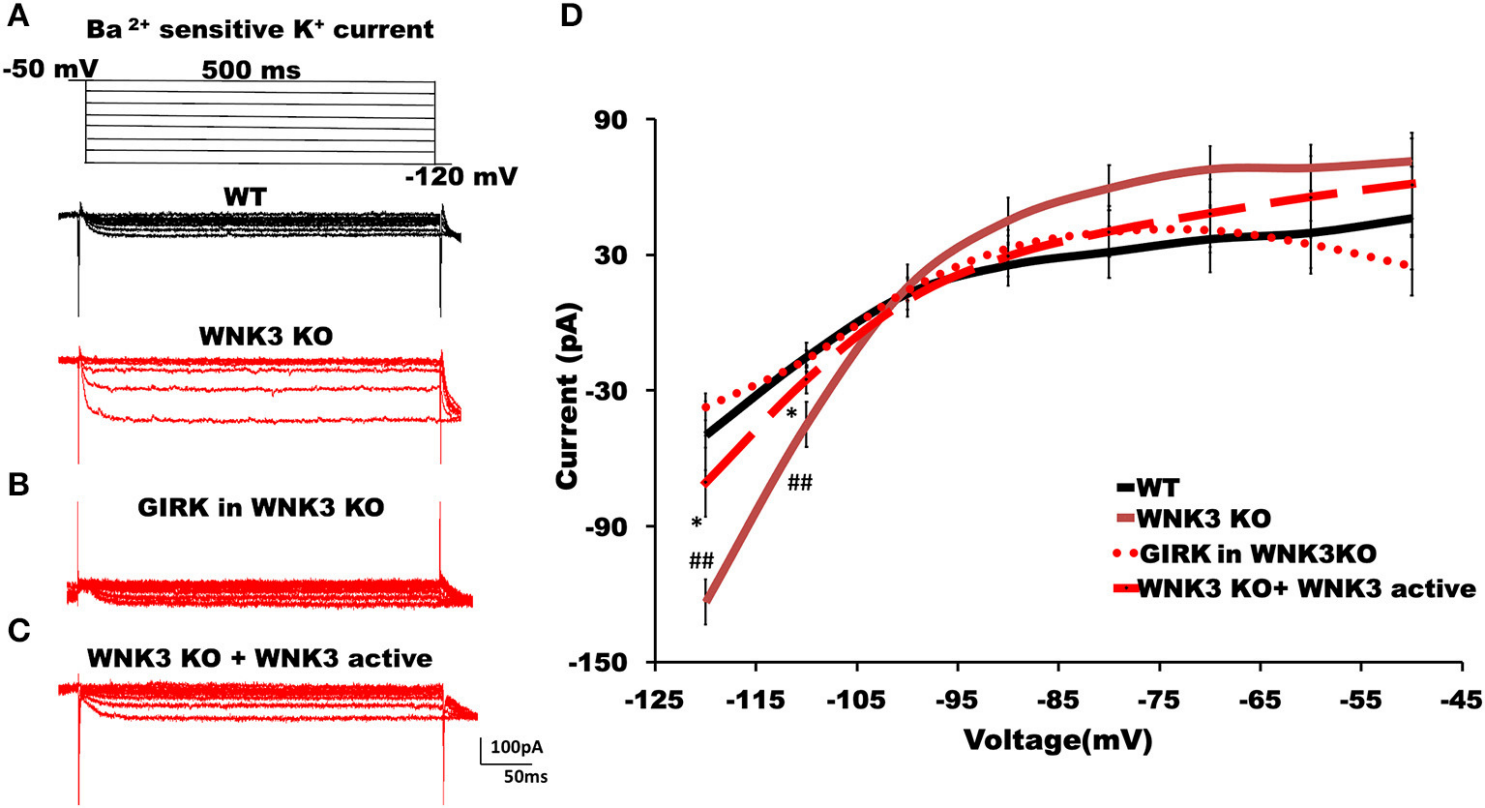
WNK3 kinase regulates classical inwardly rectifying potassium channels through kinase activity. **(A)** Representative traces of Ba^2+^-sensitive inward rectifying potassium (IRK) currents from layer V pyramidal neurons of the mPFC. Upper panel illustrates the voltage clamp protocol. WT neuron (black), WNK3 KO neuron (red), **(B)** Weak GIRK-mediated K^+^ currents in WNK3 KO neuron(red) isolated after the application of GABA_B_ receptor antagonist CGP55845 (3μM). **(C)** Reduction of IRK currents in WNK3 KO neuron following the introduction of WNK3 active fragment in the internal solution. Scale bar as shown in inset (100 pA, 50 ms). **(D)** I-V relationship of IRK currents recorded from layer V pyramidal neurons in the mPFC. Plots of mean IRK currents at different voltages are labeled as follows: WT (solid black line; *n* = 11 cells, 4 mice); WNK3 KO (solid red line; *n* = 12 cells, 4 mice); CGP55845-sensitive GIRK in WNK3 KO (dotted red line; *n* = 12 cells, 4 mice); and WNK3 KO+ WNK3 active (dashed red line; *n* = 10 cells, 4 mice). Data are represented as mean ± SEM (*K-W test, post-hoc stepwise stepdown method*. ^*##*^*P* < *0.01*, *P < *0.05*).

The hyperpolarized RMP values indicate a possible increase in the leak and inward rectifying K^+^ conductance (IRK) in WNK3 KO neurons. We therefore explored this possibility further by performing voltage-clamp recordings to measure IRK. The Ba^2+^-sensitive IRK component was isolated by subtracting current responses after the Ba^2+^ block (200 μM) from basal currents. The representative Ba^2+^-sensitive IRK recorded from WT and WNK3 KO neurons are shown in Figure 4A. Notably, the evoked currents are larger in WNK3 KO neurons. The current-voltage relationships (I-V plot) indicate a significant enhancement of inward rectification in WNK3 KO neurons (Figure 4D). This enhancement of IRK currents explains the hyperpolarized RMP values in these neurons. The layer V pyramidal neurons in the mPFC are previously reported to express GABA_B_-activated G protein-gated inwardly rectifying potassium channels (GIRK) (Takigawa and Alzheimer, 1999). Evidence also suggests that GABA_B_ receptor-dependent regulation of network excitability of mPFC (Wang et al., 2010) is important in limiting network upstates. A recent report using an ASD mice model showed hyperexcitability due to a reduction of GIRK currents (Bassetti et al., 2020). We therefore investigated the possibility of GABA_B_ receptor-mediated enhancement of GIRK currents in the WNK3 KO mice. We blocked GABA_B_ receptors by the application of CGP55845 (3 μM) and isolated the CGP-sensitive GIRK current from WNK3 KO neurons. The representative GIRK currents are shown in Figure 4B. The I-V plot of isolated GIRK currents from WNK3 KO neurons rules out the role of GIRK channels as mediators of enhanced inward rectification (Figure 4D). The WNK3 KO neurons exhibit large current amplitudes at more negative holding potentials, suggesting the enhancement of K^+^ conductance through classical inward rectifier channels (Kir 2.X). WNK3 being a kinase, we next investigated the possibility of its kinase function in the regulation of IRK in these neurons. We included an active fragment of WNK3 with intact kinase function into the internal solution and recorded IRK currents from WNK3 KO neurons. The representative Ba^2+^-sensitive current traces are shown in Figure 4C. Quantification of observed responses indicates a significant reduction of IRK conductance in these neurons (Figure 4D), suggesting WNK3 kinase-dependent regulation of Kir channels. Together, our results indicate an important role of WNK3 kinase in the maintenance of intrinsic excitability of layer V pyramidal neurons in the mPFC.

### WNK3 and KCC2 in tandem regulate classical Kir and leak K^+^ channel membrane expression

**Figure 5:**
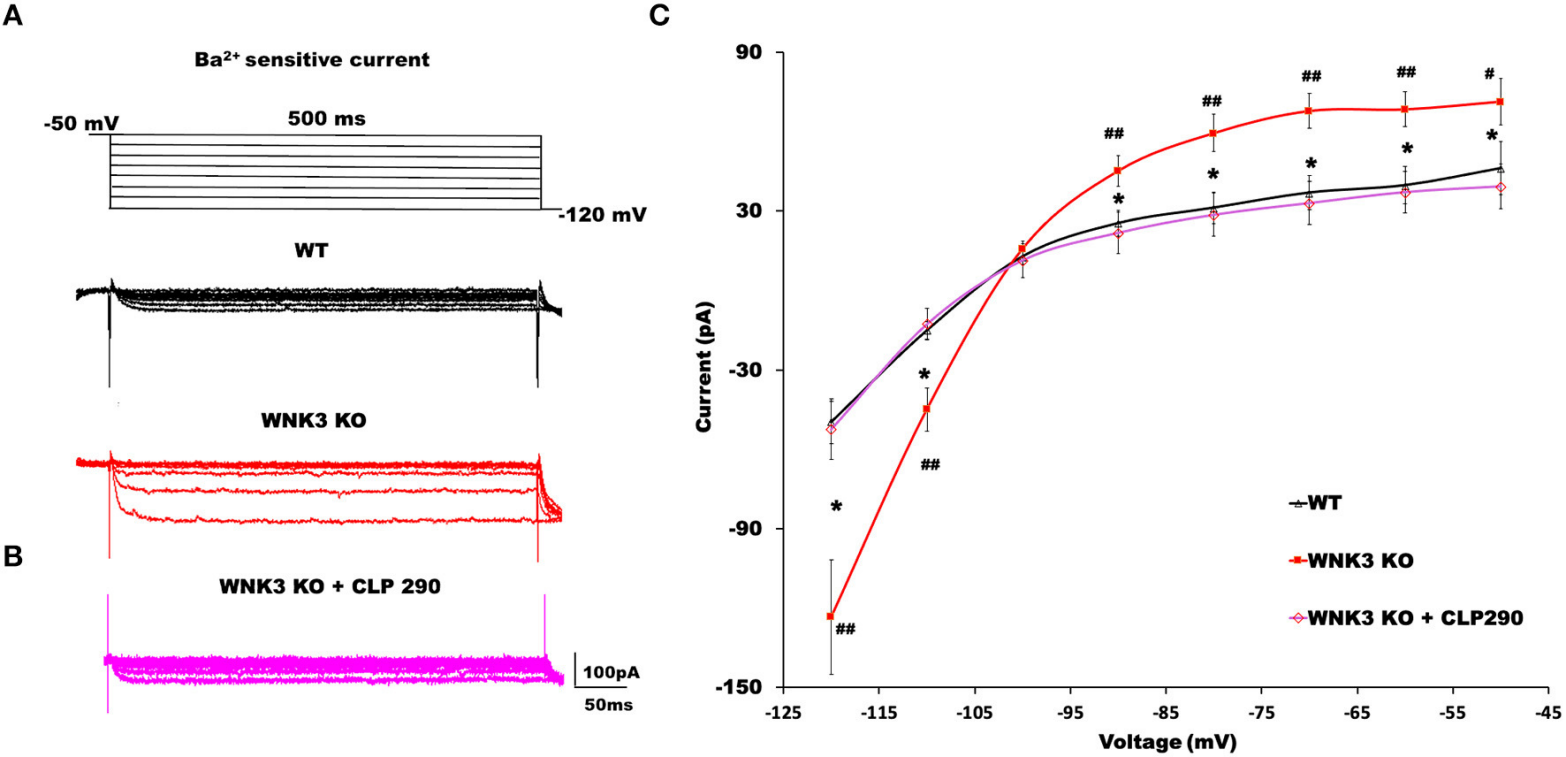
KCC2 activator CLP290 reverses increased IRK currents in WNK3 KO layer V pyramidal neurons. **(A)** Representative traces of Ba^2+^-sensitive inward rectifying potassium (IRK) currents from layer V pyramidal neurons of the mPFC. Upper panel illustrates the voltage clamp protocol. WT neuron (black), WNK3 KO neuron (red). **(B)** Representative trace of Ba^2+^-sensitive inward rectifying potassium (IRK) currents from WNK3 KO layer V pyramidal neurons after preincubation with CLP 290 (magenta). **(C)** I-V relationship of IRK currents recorded from layer V pyramidal neurons in the mPFC. Plots of mean IRK currents at different voltages are labeled as follows: WT (solid black line; *n* = 11 cells, 4 mice); WNK3 KO (solid red line; *n* = 12 cells, 4 mice); and WNK3 KO after preincubation with CLP290 (30 μM) (magenta; *n* = 12 cells, 4 mice). Data are represented as mean ± SEM (*ANOVA, post-hoc R-E-G-W F test*. ^*##*^*P* < 0.01, ^#^*P* < 0.05, ^*^P < 0.05).

The RMP is an important determinant in the driving force of GABAergic inhibition or excitation. A recent finding suggests knockdown of KCC2 reduced the membrane expression of a two-pore leak (TASK-3) K^+^ channels (Goutierre et al., 2019) depolarizing both E_GABA_ and RMP. Though we observed a depolarized E_GABA_ (Figures 1B,C), RMP was hyperpolarized (Figure 3B) in WNK3 KO neurons. We therefore next investigated the possible link between KCC2 and IRK conductance in pyramidal neurons. We blocked KCC2 activity in WT neurons by including KCC2 antagonist DIOA (30 μM) in the internal solution and recorded Ba^2+^-sensitive K^+^ currents. DIOA at this concentration has multiple off-target effects and therefore is not an ideal KCC2 antagonist. However, as a previous report observed, at reduced time scales (< 1h), it acts primarily as a KCC2 antagonist (Pellegrino et al., 2011). The representative current traces and their I-V characteristic are shown in Supplementary Figures 1B,D. We observed that on acute antagonism of KCC2 function per se, IRK currents remain unaffected as reported earlier (Goutierre et al., 2019). We then proceeded to evaluate if the application of the KCC2 activator may affect IRK currents. For this purpose, we pre-incubated acute slices with a selective KCC2 activator CLP290 (30 μM) for 1 h before recording IRK currents from WT neurons. At this concentration, this drug increases KCC2 expression, and it has been suggested that this action may be dependent on the slightly enhanced phosphorylation levels of Ser-940 of KCC2 observed after treatment (Sullivan et al., 2021). Interestingly, the CLP290 treatment produced a weak but significant enhancement of IRK currents in WT neurons (Supplementary Figures 1C,D). Similarly, experiments combining pre-incubation with CLP290 followed by IRK recordings from KO neurons were performed. The representative IRK current traces are shown in Figure 5B. We observed that instead of showing further enhancement, the recorded, IRK currents exhibited a complete reversal receding to basal current levels comparable to those recorded from WT neurons (Figure 5A). Thus, after enhancing KCC2 membrane stability with CLP290 application, normalization of IRK conductance I-V relationship was evidenced (Figure 5C). These results indicate that WNK3 and KCC2 are involved in the regulation of membrane expression of both Kir and leak K^+^ channels in layer V pyramidal neurons of mPFC.

### Loss of WNK3 reduces intrinsic excitability of layer V pyramidal neurons in the mPFC

**Figure 6:**
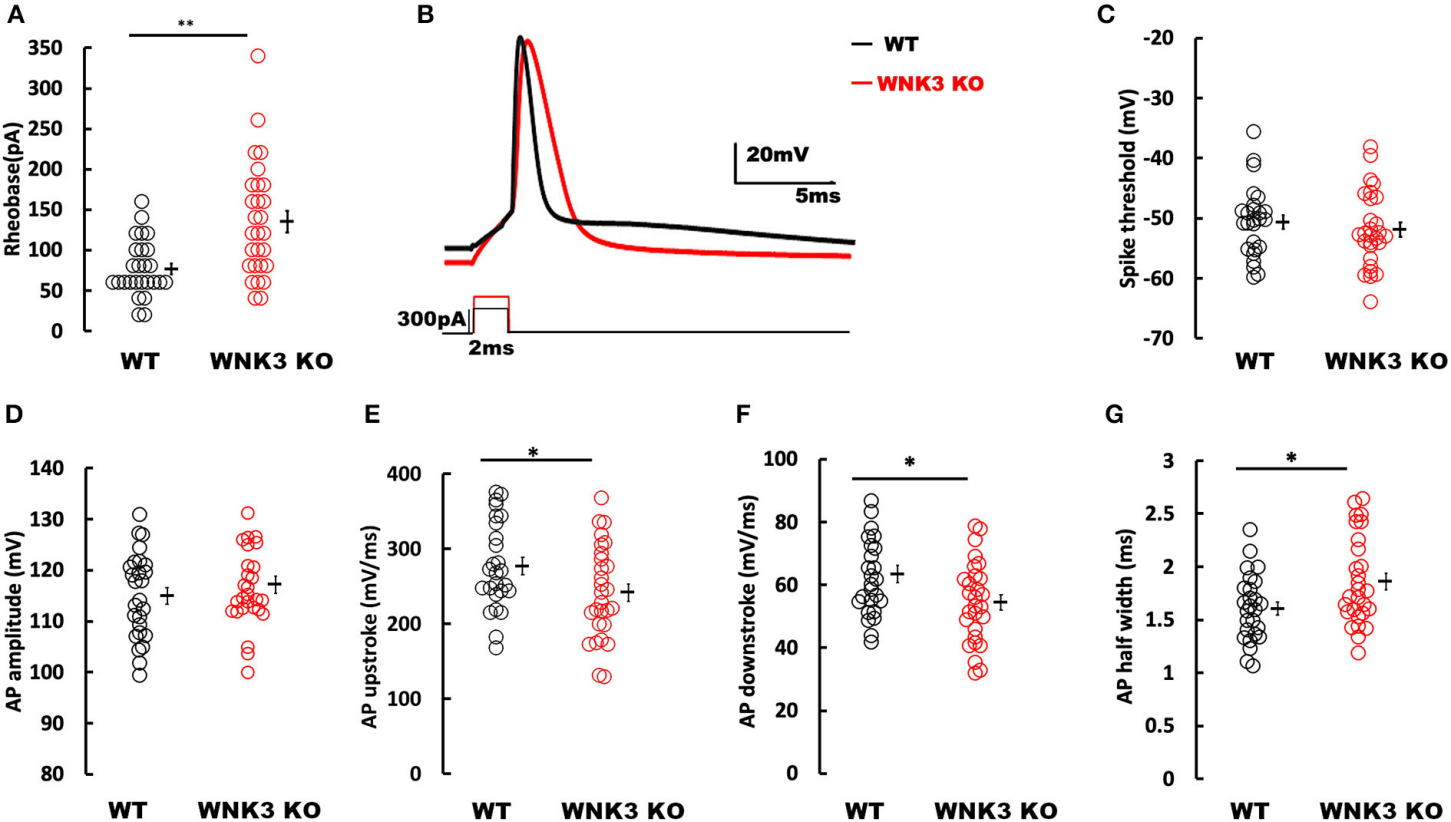
Loss of WNK3 reduces intrinsic excitability and increases single action potential (AP) duration of layer V pyramidal neurons in the mPFC. **(A)** Quantitative analysis of rheobase currents. Rheobase currents from layer V pyramidal neurons are significantly increased in the WNK3 KO group when compared to the WT group (black, WT = 76.92 ± 6.8pA, *n* = 26; red, WNK3 KO = 135 ± 13.4 pA, *n* = 28; *Mann–Whitney U* test, **P < 0.01). **(B)** Representative single AP waveforms elicited by a short pulse (2 vms) protocol (black, WT; red, WNK3 KO). Note that both the rise slope and decay slope are slower in WNK3 KO, leading to an increase in AP half-width. Scale bar as shown in inset (20 mV, 5 ms). **(C)** Quantitative analysis of AP threshold voltages shows no significant differences within groups (WT = −50.61 ± 1.14 mV; WNK3 KO = −51.93 ± 1.18 mV; *t-*test*, ns*). **(D)** AP amplitudes are not significantly different between groups (WT =114.98 ± 1.64 mV; WNK3 KO = 117.16 ± 1.66 mV; *t-*test*, ns*). **(E)** AP upstrokes are significantly slower in WNK3 KO neurons (WT = 276.85 ± 11.53 mV/ms; WNK3 KO = 241.52 ± 11.78 mV/ms; *t-*test, *P < 0.05). **(F)** AP downstrokes are significantly slower in WNK3 KO neurons (WT = 63.42 ± 2.78 mV/ms; WNK3 KO = 54.49 ± 2.42 mV/s; *t-*test, *P < 0.05). **(G)** AP half-maximal widths are significantly prolonged in the WNK3 KO group (WT = 1.61 ± 0.06 ms; WNK3 KO = 1.86 ± 0.08 ms; *t-*test, *P < 0.05). Individual data points are plotted as WT (black open circles) and WNK3 KO (red open circles). Mean values are indicated with solid bars next to the data points. Error bars represent SEM.

Following the elucidation of WNK3 action on the regulation of membrane excitability, we evaluated its effect on action potential firing in these neurons. We observed that the mean rheobase currents were significantly higher for WNK3 KO neurons (WT = 76.92 ± 6.8 pA, *n* = 26; WNK3 KO = 135 ± 13.4 pA, *n* = 28; *P* = 0.001; Figure 6A). Thereafter, the effect of WNK3 loss on AP waveform was analyzed from a single action potential (AP) elicited by a short pulse (2 ms) protocol. The representative traces for the AP waveform are illustrated in Figure 6B. Phase-plane analysis revealed no clear differences in threshold voltages at which AP was triggered (WT = −50.61 ± 1.14 mV; WNK3 KO = −51.93 ± 1.18 mV; *P* =0.424; Figure 6C). The mean amplitude of AP was observed to be similar between groups (WT = 114.98 ± 1.64 mV; WNK3 KO = 117.16 ± 1.66 mV; *P* = 0.356; Figure 6D). In addition, both upstroke (WT = 276.85 ± 11.53 mV/ms; WNK3 KO = 241.52 ± 11.78 mV/ms; *P* = 0.037; Figure 6E) and downstroke (WT = 63.42 ± 2.78 mV/ms; WNK3 KO = 54.49 ± 2.42 mV/s; *P* = 0.018; Figure 6F) of AP waveform were significantly slower in WNK3 KO neurons. As a consequence, mean duration of AP half-width was significantly prolonged in WNK3 KO neurons (WT = 1.61 ± 0.06 ms; WNK3 KO = 1.86 ± 0.08 ms; *P* = 0.015; Figure 6G). Thus, loss of WNK3 function significantly reduced intrinsic excitability of layer V pyramidal neurons.

### Repetitive firing frequencies are not affected in WNK3 KO neurons at multiples of rheobase currents

**Figure 7:**
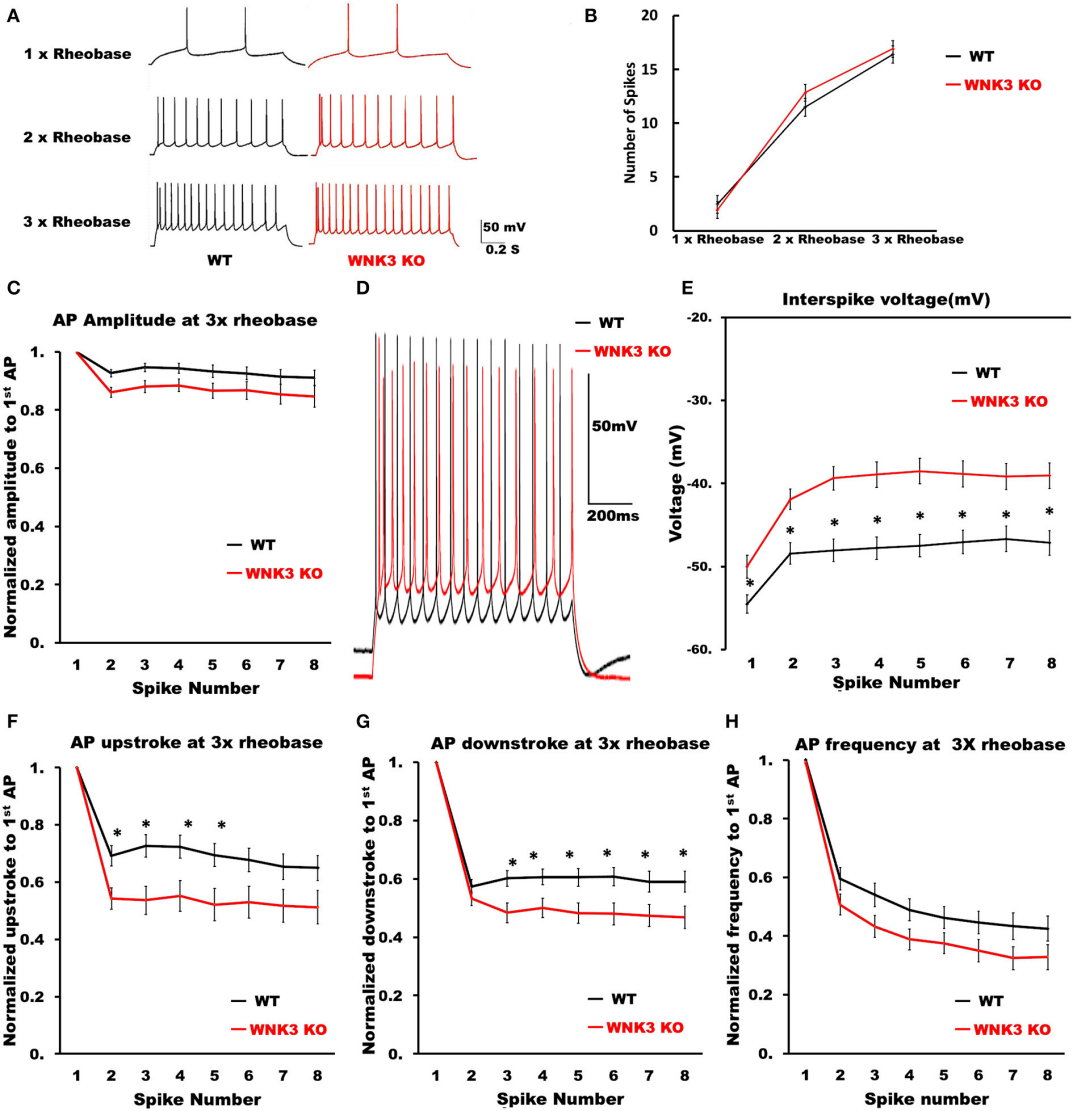
Repetitive firing frequencies are not affected in WNK3 KO neurons at multiples of rheobase currents. **(A)** Representative traces for repetitive firing at 1X, 2X, and 3X rheobase currents. WT (black) and WNK3 KO (red). The current injection duration is 1,000 ms. Scale bar as in figure inset (50 mV, 200 ms). **(B)** Plot of the relationship between the number of spikes and current injection at 1X, 2X, and 3X rheobase currents; WT (black) and WNK3 KO (red). **(C)** Normalized to first AP amplitude for first eight spikes at 3X rheobase. **(D)** Representative repetitive firing traces for 1,000 ms current injection at 3X rheobase currents for layer V pyramidal neurons (WT, black and WNK3 KO, red). Note the persistent depolarized interspike voltage in WNK3 KO neuron. Scale bar as in figure inset (50 mV, 200 ms). **(E)** Quantitative analysis of interspike voltage at 3 X rheobase (*t-*test, *P < 0.05). **(F)** Normalized AP upstroke for the first eight spikes at 3X rheobase indicates a significant reduction in upstroke during repetitive firing (*t-*test, *P < 0.05). **(G)** Normalized AP downstroke at 3X rheobase is significantly slower (*t-*test, *P < 0.05). **(H)** However, normalized instantaneous frequencies at 3X rheobase are not affected.

We then examined the repetitive firing and its adaptation parameters following 1 s long step current injections. In view of the significant reduction in input resistance and increase of rheobase currents in WNK3 KO neurons, the input-output relationship was significantly reduced (data not shown). Therefore, to estimate if the neuronal gain was affected, we compared AP firing frequencies at multiples of rheobase current injections (1x, 2x, and 3x rheobase). The repetitive firing patterns at these current injections are illustrated in Figure 7A. Statistical analysis revealed no significant differences between WT and WNK3 KO neurons, suggesting no effect of WNK3 loss on neuronal gain (Figure 7B). The assessment of repetitive firing and adaptation was performed at three times rheobase current. The comparison between amplitudes of the first eight spikes normalized to the peak amplitude of the first AP showed no significant difference between WT and WNK3 KO groups (Figure 7C). In addition, interspike voltages (ISV) were found to be significantly depolarized in WNK3 KO neurons (Figures 7D,E). Also, comparisons of AP upstroke and downstroke revealed significant decrements in WNK3 KO neurons (Figures 7F,G). Sluggish upstroke and downstroke of spikes would predict a reduction in the frequencies of spikes. Instead, interestingly, the comparison between normalized instantaneous frequencies (Figure 7H) and firing frequencies (Figure 7B) showed no significant differences between WT and WNK3 KO neurons.

### Loss of WNK3 kinase leads to simultaneous reduction of excitatory inputs and increment of inhibitory inputs to layer V pyramidal neurons

**Figure 8:**
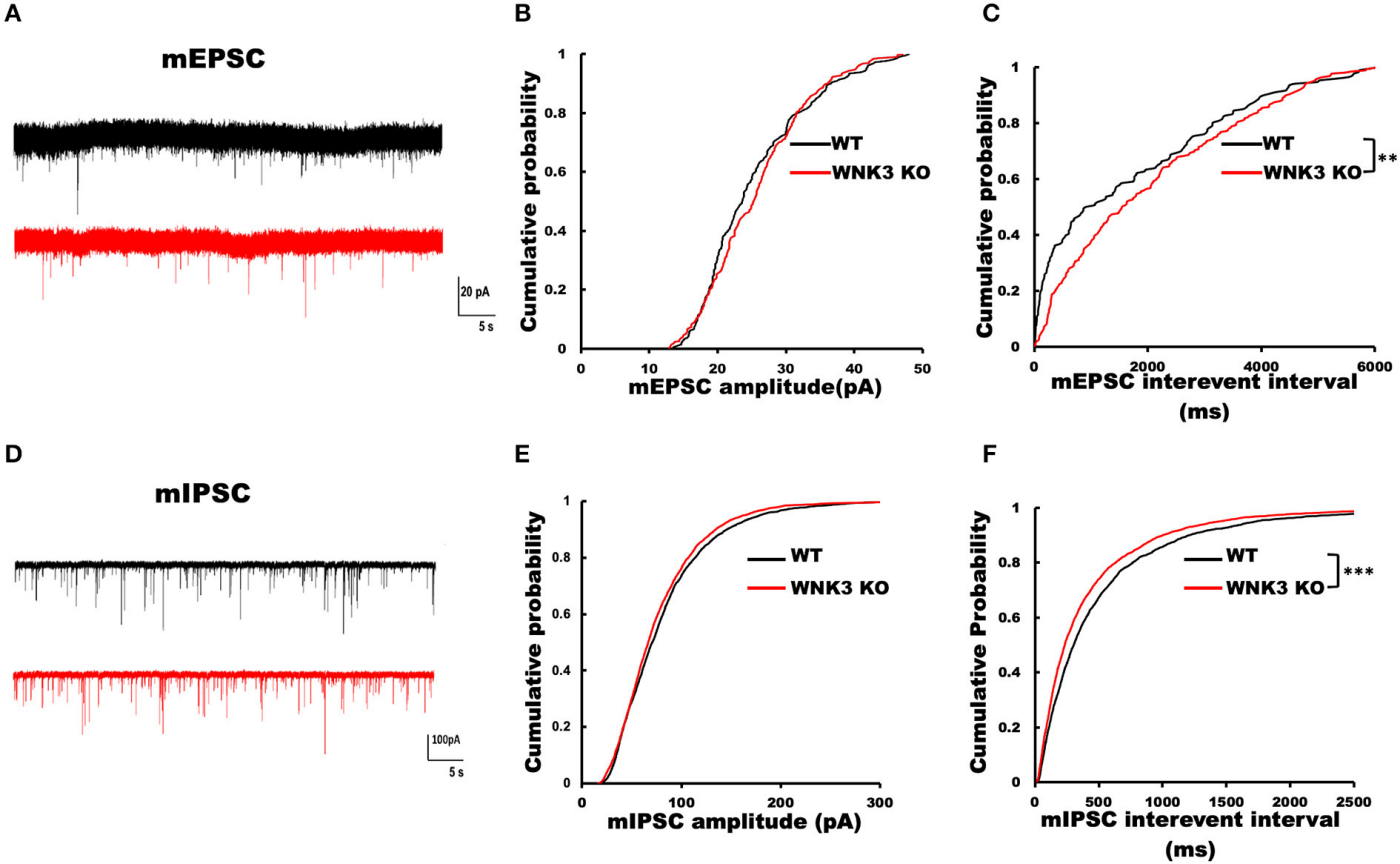
Loss of WNK3 kinase leads to simultaneous reduction and increment of excitatory and inhibitory inputs, respectively, to layer V pyramidal neurons in the mPFC. **(A)** Representative mEPSC current traces recorded from layer V pyramidal neurons, WT (black) and WNK3 KO (red). Holding voltage was set at −70 mV with a CH_3_SO_3_K-based normal Cl^−^ internal solution (see Methods section for composition). Scale bar as in figure inset (20 pA, 5 s). **(B)** Cumulative probability plot of amplitude distribution of mEPSC events. **(C)** Cumulative probability plot of interevent intervals (IEIs) of mEPSC events, indicating a reduction in the frequency of mEPSC recorded from WNK3 KO neurons (*N* = 24 cells; *K-S test*, **P < 0.01). **(D)** Representative mIPSC current traces recorded from layer V pyramidal neurons, WT (black) and WNK3 KO (red). Holding voltage was set at −70 mV with a 150.0 mM CsCl-based solution (see Methods section for composition). Scale bar as in figure inset (100 pA, 5 s). **(E)** Cumulative probability plot of the amplitude distribution of mIPSC events. **(F)** Cumulative probability plot of IEI of mIPSC events. Note the leftward shift of IEI cumulative distribution of mIPSC events recorded from WNK3 KO neurons clearly indicating an increase in mIPSC frequency (*N* = 25 cells; *K-S* test, ***P < 0.001). Cumulative plots are color coded as WT (black) and WNK3 KO (red).

To understand the role of WNK3 in the development of neural circuits in the mPFC, we performed recordings for both excitatory and inhibitory miniature postsynaptic currents (mEPSCs and mIPSCs) from layer V pyramidal neurons. The representative mEPSC current traces recorded from WT and WNK3 KO neurons are shown in Figure 8A. Analysis of mEPSC event distributions for amplitude and interevent intervals was restricted to events within 50 pA. Comparisons of mEPSC amplitude distributions show no differences between WT and WNK3 KO neurons (*P* = 0.1516, *n* = 24; Figure 8B). The distribution of interevent intervals was significantly right-shifted for WNK3 KO neurons, indicating a decrease in the frequency of mEPSC events (*P* = 0.0046, *n* = 24; Figure 8C). On the other hand, evaluation of mIPSC events revealed a contrasting phenomenon. The representative mIPSC recordings for each group are shown in Figure 8D. The comparison between the distribution of mIPSC interevent intervals showed a significant leftward shift for WNK3 KO neurons, indicating the enhanced frequency of mIPSCs (*P* = 0.000001, *n* = 25; Figure 8F). The mIPSC amplitudes were apparently affected in the cumulative distribution plots (*P* =0.0002, *n* = 25; Figure 8E).

### WNK3 KO mice exhibit normal locomotion and social behavior but deficits in prepulse inhibition

**Figure 9:**
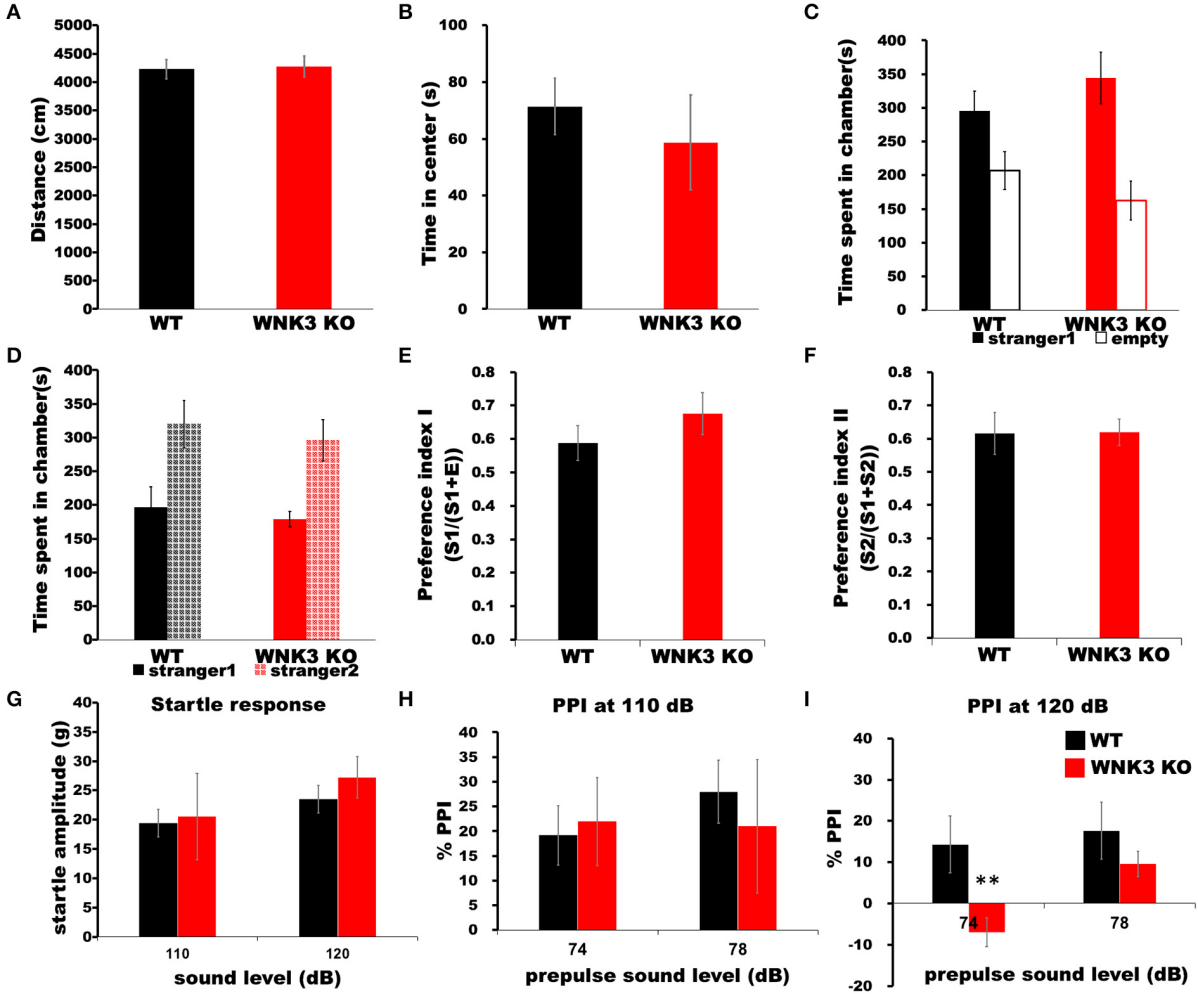
WNK3 KO mice exhibit normal locomotion and social behavior but deficits in prepulse inhibition. **(A)** Open field test show no difference in total distance traveled (WT = 4,229.71 ± 170.84 cm, *n* = 8 mice; WNK3 KO = 4,271.45 ± 186.62 cm, *n* = 4 mice) indicating normal exploratory and locomotor activity. **(B)** Time spent in the center of the open field arena is similar between WT and WNK3 KO mice indicating anxiety levels are similar (WT = 71.39 ± 8.82 s; WNK3 KO = 58.73 ± 6.13 s). **(C)** No alterations in sociability were observed between WT and WNK3 KO mice. **(D)** Social novelty recognition was not affected in WNK3KO. Preference indices for **(E)** social interaction and **(F)** social memory were also not affected by WNK3 deletion. **(G)** Startle response amplitudes at 110 dB and 120 dB were not different between groups **(H)**. Percent PPI at 110 dB to prepulse of 74 and 78 dB was not affected. **(I)** Percent PPI at 120 dB to prepulse of 74 dB was significantly reduced in WNK3 KO (*t-*test **P < 0.01) and with the extent of PPI showing reduction at 78 dB does not reach significance.

Finally, we assessed the impact of a decrease in neuronal excitability and alterations on mPFC cortical circuits during development by evaluating behavioral changes in WNK3 KO mice. The time spent in the center of the arena and total distance traveled within a period of 5 min in an open-field apparatus were not significantly different between WNK3 KO and WT littermates, indicating no changes in anxiety levels (Figure 9A) and locomotor activities, respectively (Figure 9B). No alterations in sociability (Figure 9C) and social novelty (Figure 9D) were observed, resulting in similar preference indices for social interaction (Figure 9E) and social memory (Figure 9F) between groups. We also evaluated startle response and prepulse inhibition (PPI), a measure of sensorimotor gating (Swerdlow and Geyer, 1998), in these mice. Interestingly, while startle response was unchanged (Figure 9G), we observed a deficit in PPI response after a prepulse of 74 dB in WNK3 KO mice at 120 dB (Figure 9I) but not at 110 dB (Figure 9H), which showed no significant differences. These results indicate a deficit in sensorimotor gating in WNK3 KO mice without compromising social behavior. In addition, locomotion and anxiety levels are unaffected by the loss of WNK3 function.

## Discussion

In the present study, using a constitutive WNK3 KO mouse, we investigated the role of WNK3 kinase in brain development. In particular, we examined its role in the regulation of chloride homeostasis in pyramidal neurons, its function in the control of neuronal excitability, and the effect on the development of neural circuits governing synaptic connectivity in the mPFC. Furthermore, we analyzed the behavioral impact of changes on brain development manifested by WNK3 loss. Our findings indicate a significantly depolarized reversal potential for GABA_A_ receptor-mediated currents by 6 mV at P 21. This corresponded to higher resting [Cl^**−**^]_i_ level of ~4 mM in KO mice than in WT littermates. However, expected alterations in the phosphorylation levels of the WNK1-SPAK/OSR1 signaling cascade and their downstream targets NKCC1 and KCC2 were not observed in KO mice. Meanwhile, the RMP of neurons was more hyperpolarized by 7 mV, and the rheobase current necessary for firing induction was increased in KO mice. These were due to an increase in IRK currents. Investigations ruled out the possibility of increased activation of GIRK-mediated reduction in neuronal excitability; instead, this reduction was mediated by Kir channels. Interestingly, supplementation of an active form of WNK3 with intact kinase function reversed these changes. We also observed a novel association between KCC2 and WNK3 affecting the IRK currents mediated by Kir and leak K^+^ channels suggestive of their important role in the regulation of the RMP. Analysis of individual action potential spikes indicated prolonged half widths in KO neurons. Evaluation of repetitive action potential firing at three times rheobase current surprisingly showed no effect on instantaneous firing frequencies and neuronal gain. Moreover, synaptic current recordings from KO neurons revealed that the frequency of mEPSCs was reduced, whereas those of mIPSCs were increased. These developmental changes in membrane and synaptic properties of pyramidal neurons in unison with yet-to-be elucidated changes in other cellular subtypes culminated as deficits in PPI, a measure of sensorimotor gating involving multiple brain regions including the mPFC, in WNK3 KO mice.

The primary physiological role ascribed to the WNK-SPAK/OSR1 signaling cascade is its maintenance of chloride homeostasis by phosphorylation-dependent regulation of cation-Cl^−^co-transporters (Alessi et al., 2014). In neurons, [Cl^−^]_i_ levels are critical for determining GABA action (Ben-Ari, 2002) and cell volume regulation (Akita and Okada, 2014; Huang et al., 2019). Our gramicidin-perforated patch-clamp recordings of layer V pyramidal neurons from WNK3 KO mice at P21 revealed average E_GABA_ values depolarized by 6 mV (Figure 1C). This corresponds to a weak increase in resting [Cl^−^]_i_ levels by 4 mM. However, this enhancement was not paralleled by an increase in WNK1-SPAK/OSR1 signaling, because no changes in activated pWNK1, pSPAK, and pOSR1 protein levels were observed (Figure 1). The pNKCC1 levels were also not affected, ruling out an indirect effect on the NKCC1 Cl^−^ import function. We also investigated if there is a more pronounced effect of WNK3 loss of function on the phosphorylation level of Thr1007 residue in the C-terminal domain of KCC2, which may explain the elevated resting [Cl^−^]_I_ levels. However, we observed no significant differences when compared to the WT group (Figure 2). Our observations are not in agreement with a recent report (Lim et al., 2021). In this study, employing a primary hippocampal neuronal culture, a knockdown of WNK3 using lentiviral-mediated shRNA at DIV 1 was established, a time point at which the fetal phase of development nears completion. This produced a reduction in the WNK3 protein levels between 60 and 80% of control levels. This also reduced the pThr1007 KCC2 levels and contributed to a hyperpolarized E_GABA_. These findings fit the dogma that loss of WNK-SPAK/OSR1 regulation would facilitate the enhancement of GABAergic inhibition. However, whether loss of WNK3 leads to regulation of WNK1 activity by changes in [Cl^−^]_i_ as predicted earlier (Heubl et al., 2017) was not explored. If one were to make a reasoned argument, the main difference between our approach and that of Lim et al. (2021) is that we used constitutive KO of *WNK3* in contrast to a knockdown approach with a reported 60–80% efficiency. This may contribute to differences. It would be noteworthy that high expression of WNK3 in the fetal developmental phase, as reported by Küry et al. (2022), would remain unaffected in the knockdown model. In contrast, a constitutive KO would affect both phases of brain development. The WNK-SPAK/OSR1 cascade is one of the regulatory pathways affecting the downstream functions of both NKCC1 and KCC2, and their disruption may trigger an alteration in other regulatory pathways. The KCC2 function-mediated lowering of [Cl^−^]_i_ in mature neurons is subject to regulation by multiple mechanisms controlling its membrane expression and stability (Côme et al., 2019). Furthermore, changes in phosphorylation-dependent posttranslational regulation of KCC2 at other amino acid residues in its C-terminal region are quite possible. In addition to these possibilities, Cl^−^ movement across the neuronal membrane is also facilitated by Ca^2+^-activated Cl^−^ channel (TMEM6B), volume-regulated anion channels (VSOR), and a novel voltage-gated Cl^−^ channel (SLC26A11) (Akita and Fukuda, 2020). If the WNK3 function entails regulation of any of these Cl^−^ channels, the weak enhancement of [Cl^−^]_i_ remains a possibility in the WNK3 KO mice. More importantly, the functional impact of WNK3-mediated regulation of [Cl^−^]_i_, independent of activation of WNK1-SPAK/OSR1 cascade, would require careful examination.

The membrane properties of layer V pyramidal neurons rapidly change during the second postnatal week with adult-like properties observed by P21 (Zhang, 2004). In our mouse model, WNK3 deletion led to multiple changes in membrane excitability. The RMP was significantly hyperpolarized. In addition, input resistance values were significantly reduced. During normal development, the decrease in input resistance leading to deeper RMP between P14 and P21 is attributed to the enhancement of leak K^+^ conductance mediated by KCNK channels (Goldstein et al., 2001). Incidentally, expression of TWIK1 and TASK-3, two-pore leak K^+^ channels, increases during this phase (Aller and Wisden, 2008). Previous recordings from layer V pyramidal neurons of the mouse mPFC observed two distinct K^+^ conductance systems attributed to leak K^+^ channels and IRK channels (Day et al., 2005). These neurons are reported to express a GABA_B_ receptor-dependent GIRK conductance as well (Takigawa and Alzheimer, 1999). To confirm the enhancement of resting K^+^ currents in KO neurons, the Ba^2+^-sensitive inward K^+^ current component was isolated by subtracting current responses after Ba^2+^ block (200 μM) from basal currents (Figure 4). The K^+^ currents were larger in WNK3 KO neurons confirming our hypothesis. Furthermore, these currents showed strong inward rectification at voltages deeper than the E_K_ value of−103 mV ruling out the enhancement of leak conductance which is characterized by linear I-V characteristics (Goldstein et al., 2001; Day et al., 2005). In addition, isolated GIRK currents were minimal (~37.2 pA; GIRK vs ~ 123.5 pA; total IRK at −120mV) in comparison to total IRK currents recorded in WNK3 KO neurons. The observed small peak amplitudes of GIRK currents at P21 are in agreement with previous results which show enhancement of GABA_B_-dependent GIRK conductance in rodent mPFC only after P25-P36 (Wang et al., 2010; Bassetti et al., 2020). Thus, the observed strong inward rectification in WNK3 KO neurons is ascribed to enhanced classical inward rectifier channel function (Kir 2.X) that is expressed in these neurons (Day et al., 2005). The classical inward rectifier channels are regulated by multiple mechanisms (see review Hibino et al., 2010). More recent reports have shown that the WNK-SPAK/OSR1 cascade also plays a regulatory role in Kir channel functions. Both WNK1 and WNK4 in a kinase-independent manner reduce the surface expression of ROMK (Kir1.1) channels (Liu et al., 2009). Another report finds that activated OSR1 increased membrane stabilization of specific Kir channel subtypes (Kir2.1 and Kir2.3) (Taylor et al., 2018). However, till now, there are no reports suggesting direct regulation of inward rectification in neurons by WNK3. Based on our loss of function and rescue experiments with kinase-active WNK3 fragment in the internal solution, we can confirm that normal WNK3 activity would reduce the strong inward rectification. This result implies that endogenous WNK3 may either directly phosphorylate classical Kir channels attenuating IRK currents in pyramidal neurons or indirectly modulate another regulatory sequence involved in the membrane expression of these channels. We therefore explored the possibility of indirect modulation of IRK currents by WNK3. It has recently been reported that only reduced membrane expression of KCC2 and not reduced transport activity led to a robust decrease in the membrane expression of two-pore leak (TASK-3) K^+^ channels in the dentate gyrus granule cells and in CA1 pyramidal neurons (Goutierre et al., 2019). The layer V pyramidal neurons in the adult rodent cortex show moderate levels of TASK-3 transcripts when compared to granule cells (Talley et al., 2001), suggesting a lesser fraction of IRK currents would be mediated by leak K^+^ channels currents (Day et al., 2005). We confirmed that blocking KCC2 transporter activity per se in WT neurons did not reduce IRK currents (Supplementary Figure 1). Instead, increasing the membrane stability of KCC2 with pre-incubation with CLP290 produced a slight but significant enhancement of IRK currents, corroborating that an increase in KCC2 membrane stability enhances IRK currents (Seja et al., 2012; Goutierre et al., 2019). This linear increase may occur after changes in the membrane localization of TASK-3 channels and would require additional experiments. In stark contrast, a similar pretreatment with CLP290 in WNK3 KO neurons produced a reversal of IRK currents to levels similar to WT neurons (Figure 5). This would indicate that the enhanced IRK currents are mediated by a probable increase in classical Kir channel membrane expression in WNK3 KO neurons, in addition to the fact that WNK3 kinase activity would depend on KCC2 membrane stability or vice versa. Delving deeper, it might also be speculated that WNK3 plays a role in the interaction between KCC2 and TASK-3 channels. Conversely, an increase in KCC2 membrane expression inhibits classical Kir channel membrane expression, but increases TASK-3 channel membrane expression. In addition, our result could also imply that constitutive WNK3 loss may reduce the membrane stability of KCC2 by mechanisms other than regulation of pThr1007 KCC2 levels. This could also explain the weak increase in resting [Cl^−^]_i_ levels in WNK3 KO neurons; however, confirmation of this assumption would require further investigation. Overall, in the context of regulation of IRK currents in neurons, among the different possible mechanisms, we hypothesize that WNK3 and KCC2 in tandem regulate the expression of Kir channels and leak K^+^ channels (TASK-3 channels), thus maintaining their relative proportions in the membrane. This regulating cascade would possess the wherewithal to control the RMP and thereby act as a restraint to exaggerated shifts in the driving force of Cl^−^. Furthermore, it would be interesting to examine how different pathological conditions affect this regulatory mechanism. Our findings therefore assign a novel function to WNK3 in tandem with KCC2 in the regulation of RMP and thereby neuronal excitability.

The waveform analysis of single AP elicited by a short pulse protocol indicates no differences in AP threshold voltage and single AP amplitude between the genotypes. The observed reduction in input resistance and membrane time constant (Figure 3) due to the enhancement of IRK currents (Figure 4) explains the significant increase in rheobase currents (Figure 6) recorded in KO neurons. Our analysis of a single AP waveform shows slower AP upstroke and downstroke prolonging the AP duration. The slower AP decay in KO neurons suggests changes in K^+^ currents during the repolarization phase. During the sustained neuronal activity, as in repetitive firing, these effects are accentuated as significant decrements in AP downstrokes and relatively slower upstrokes in KO neurons (Figures 6E,F). This reduction would predict greater adaptation in KO neurons. However, surprisingly, no effects on firing frequencies were observed in KO neurons (Figures 7B,H). The repolarization of spikes in pyramidal neurons is primarily contributed by the delayed rectifier K^+^ channels (K_**V**_2) forming heteromeric channels in association with other K_V_ channels (Misonou et al., 2005). However, for these channels to maintain activity, sufficient membrane depolarization for activation is required. Functionally, these assemblies contribute to maintaining repetitive firing in pyramidal neurons by regulation of the interspike trough voltages (Liu and Bean, 2014; Saitsu et al., 2015). In our findings, though the ISV levels are significantly depolarized in comparison to WT, uniform trough voltages are maintained (Figure 7). This suggests that during repetitive firing, another repolarizing conductance in concurrence mediated by delayed rectifier K^+^ channels sustains repetitive firing in the KO neurons. This may occur as a compensatory mechanism to counter changes in predicted changes in adaptation parameters or occur due to the absence of WNK3 regulating these channels. If we were to speculate, a candidate could be BK-type Ca^2+^-activated K^+^ channels. Both experimental and biophysical modeling data indicate a facilitatory role of BK channels in high-frequency firing in pyramidal neurons (Gu et al., 2007). The prolongation of spike duration as observed (Figure 6) may contribute to increased Ca^2+^ entry into the KO neurons. This enhanced [Ca^2**+**^]_i_ may affect the BK channel function. Recent evidence, utilizing artificial expression systems in HEK cells, indicated that WNK-SPAK/OSR1 cascade affects the BK channel function (Liu et al., 2015).

We further assessed the role of WNK3 in the development of cortical networks by recording mPSCs from pyramidal neurons. Our results indicate a significant reduction in excitatory inputs to layer V pyramidal neurons in KO mice (Figure 8C). On the contrary, inhibitory inputs to these neurons were enhanced (Figure 8F). Incidentally, shRNA knockdown of WNK3 showed an increase in mEPSC amplitudes from hippocampal culture neurons (Lim et al., 2021). The possible mechanisms underlying this dichotomy in the synaptic connectivity pattern between constitutive KO and knockdown of WNK3 warrants further investigation. The functional maturation and integration of distinct GABAergic interneuron subtypes in the cortical circuitry (Lim et al., 2018) also coincide with changes in WNK3 expression. In this period, both rapid modifications of layer V pyramidal neuron morphology (Zhang, 2004; Romand et al., 2011) and layer-specific synaptic inputs in the mPFC (Zhang, 2004; Kroon et al., 2019) have been observed. Moreover, circuit refinements during this period are neuronal activity-dependent (Burrone et al., 2002). In the constitutive WNK3 KO mice, the intrinsic excitability of pyramidal neurons is significantly reduced (Figure 3), which may contribute to differences in synaptic inputs when compared to WT littermates. In addition, KCC2 membrane expression is an important regulator of excitatory synapse formation (Watanabe and Fukuda, 2015). Thus, differences in KCC2 membrane stability between WT and WNK3 KO neurons, which may be implied from our results (Figure 5), also need to be considered as a probable mechanism. As the mIPSC frequencies are increased, additional experiments would be necessary to ascertain if this presynaptic change is due to enhanced intrinsic excitability of gabaergic neurons or at the level of gabaergic terminals with changes in release probability and enhancement of synaptic boutons. The gabaergic interneurons would also normally express WNK3, and it would be important to examine if interneuron-specific loss of WNK3 exhibits the same electrophysiological characteristics. In the scenario of a similar enhancement of IRK conductance in inhibitory neurons, the input resistance (**R**_**In**_) would be decreased and might affect the low threshold spiking neurons with reportedly higher **R**_**In**_ to a greater extent than mature parvalbumin-positive fast-spiking interneurons.

The cumulative effect of these developmental changes in WNK3 KO mice manifests as deficits in PPI, an indicator of abnormal information processing during sensorimotor gating (Figure 7G). Deficits in PPI are a hallmark feature in patients presenting with varied neuropsychiatric disorders, including schizophrenia (Kohl et al., 2013). The neural circuit modulating PPI is the cortico-striatal-pallido-thalamic (CSPT) circuit comprising multiple brain regions including the mPFC and hippocampus (Swerdlow and Geyer, 1998). Several reports indicate that either increase or decrease in mPFC activity leads to deficits in PPI (Tapias-Espinosa et al., 2019). In addition, disruptions in neurodevelopmental sequelae of mPFC by different strategies like isolation rearing (Day-Wilson et al., 2006) and prenatal exposure to drugs (Toriumi et al., 2016) and toxins (Wischhof et al., 2015) all produce PPI deficits. The loss of WNK3 regulation reducing neuronal excitability of pyramidal neurons in the mPFC and yet unidentified effects on other neuronal subtypes leading to observed changes in synaptic connectivity could disrupt the functional development of the mPFC, thereby affecting the CSPT circuit and resulting in PPI deficits.

It has been proposed that the downregulation of the WNK-SPAK/OSR1 signaling cascade may work as a therapeutic strategy in disorders of altered inhibition, such as epilepsy, schizophrenia, and autism spectrum disorders (Kahle and Delpire, 2016; Côme et al., 2019). Incidentally, preliminary investigations examining the effects of the rare WNK3 variants predicted to produce loss of function (Küry et al., 2022) when expressed in a HEK293T cell line reported a 60% reduction in WNK3 protein levels and reduction of pThr1007 KCC2 levels. This reduction would facilitate an increase in inhibitory tone and in all probability produce a shift in the E/I balance toward enhanced inhibition. However, interestingly, the behavioral assessments of these subjects revealed that 38% showed seizures. Some of them manifested attention deficit hyperactivity disorders (ADHD) and auto-aggressiveness with profound intellectual disabilities observed in all affected individuals. Clinically, these phenotypes are normally associated with increased excitability of brain function. These observations of functional dichotomy may hold the key to explaining the variance of our findings. All the affected individuals underwent developmental sequalae with the predicted loss of function produced by the WNK3 variants. The developmental phase of brain development is a labile period, and loss of WNK3 function may trigger parallel compensatory mechanisms possible due to the great complexity and multiple redundancies inherent in the system with some of them being beneficial and others detrimental to the long-term health of the brain manifesting as disease phenotypes. Future studies with animal models incorporating the human WNK3 variants would be essential in elucidating and reconciling these reported differences.

To conclude, our findings suggest that constitutive loss of WNK3 in pyramidal neurons plays a critical role in the maintenance of neuronal excitability by reducing resting membrane K^+^ conductance and increasing the number of excitatory synaptic inputs, with a weak effect on intracellular Cl^−^ homeostasis. Thus, the basal function of WNK3 would be the maintenance and/or development of both intrinsic and synaptic excitabilities.

## Data availability statement

The original contributions presented in the study are included in the article/Supplementary material, further inquiries can be directed to the corresponding author/s.

## Ethics statement

The animal study was reviewed and approved by Committee for Animal Care and Use (No. 2018048) Hamamatsu University School of Medicine.

## Author contributions

AS, TW, and MW performed experiments. AS, TW, MW, and YH performed analysis. AS, TA, and AF designed experiments. ES and SU generated mice and contributed with suggestions on the manuscript. AS and AF wrote the manuscript. All authors contributed to the article and approved the submitted version.

## Funding

This work was supported by Grants-in-Aid for Scientific Research on Innovative Areas (Non-linear oscillology) #15H05872 from the Ministry of Education, Culture, Sports, Science and Technology of Japan (to AF), Grants-in-Aid for Scientific Research (B) #21H02661 from the Japan Society for the Promotion of Science, and Grant-in-Aid for Transformative Research Areas (A) #21H05687 from the Ministry of Education, Culture, Sports, Science and Technology of Japan.

## Conflict of interest

The authors declare that the research was conducted in the absence of any commercial or financial relationships that could be construed as a potential conflict of interest.

## Publisher's note

All claims expressed in this article are solely those of the authors and do not necessarily represent those of their affiliated organizations, or those of the publisher, the editors and the reviewers. Any product that may be evaluated in this article, or claim that may be made by its manufacturer, is not guaranteed or endorsed by the publisher.
