# Supplementary material for: WNK3 kinase maintains neuronal excitability by reducing inwardly rectifying K+ conductance in layer V pyramidal neurons of mouse medial prefrontal cortex
Source: Front Mol Neurosci. 2022 Oct 13;15:856262. doi: 10.3389/fnmol.2022.856262 (PMC9613442; doi:10.3389/fnmol.2022.856262)
Supplement: Supplementary file 1 [file Image_1.pdf]

Supplementary Material

WNK3 kinase maintains neuronal excitability by reducing inwardly rectifying K^+ conductance in layer V pyramidal neurons of mouse medial prefrontal cortex.

Supplementary Figure

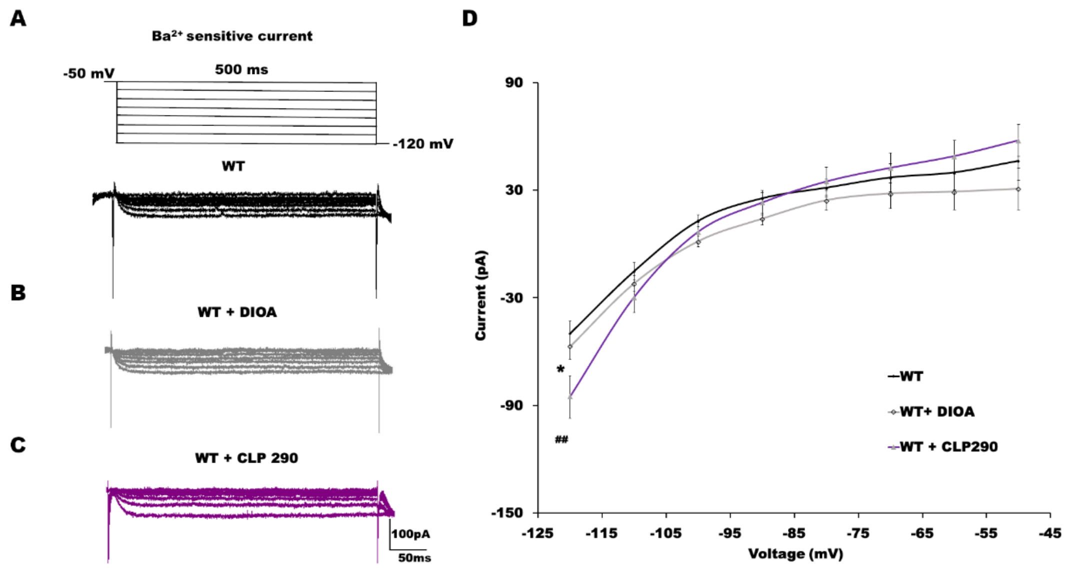

Supplementary Figure 1. KCC2 activator CLP290 increased IRK currents in layer V pyramidal neurons from WT mice (A) Representative traces of Ba^{2+}-sensitive inward rectifying potassium (IRK) currents from layer V pyramidal neurons of the mPFC. Upper panel illustrates the voltage clamp protocol. WT neuron (black), (B) Representative trace of Ba^{2+}-sensitive inward rectifying potassium (IRK) current after blocking of KCC2 activity with DIOA (30 µM) in internal solution (grey) (C) Representative trace of Ba^{2+}-sensitive inward rectifying potassium (IRK) currents from WT neurons after preincubation with CLP 290 (purple). (D) I-V relationship of IRK currents recorded from layer V pyramidal neurons in the mPFC. Plots of mean IRK currents at different voltages are labelled as follows: WT (solid black line; n = 11 cells, 4 mice); WT with DIOA (solid grey line; n = 11 cells, 4 mice); and WT

after preincubation with CLP290 (30 μM) (purple; n = 10 cells, 4 mice). Data is represented as Mean ± SEM. (*ANOVA, posthoc R-E-G-W F test. ## P< 0.01, * P<0.05*).
